# Analysis of Therapeutic Inertia and Race and Ethnicity in the Systolic Blood Pressure Intervention Trial: A Secondary Analysis of a Randomized Clinical Trial

**DOI:** 10.1001/jamanetworkopen.2021.43001

**Published:** 2022-01-10

**Authors:** Alexander R. Zheutlin, Favel L. Mondesir, Catherine G. Derington, Jordan B. King, Chong Zhang, Jordana B. Cohen, Dan R. Berlowitz, D. Edmund Anstey, William C. Cushman, Tom H. Greene, Olugbenga Ogedegbe, Adam P. Bress

**Affiliations:** 1 Department of Internal Medicine, University of Utah, School of Medicine, Salt Lake City; 2 Department of Population Health Sciences, University of Utah, School of Medicine, Salt Lake City; 3 Department of Biostatistics, School of Public Health, Boston University, Boston, Massachusetts; 4 Institute for Health Research, Kaiser Permanente Colorado, Aurora; 5 Department of Biostatistics, Epidemiology, and Informatics, Perelman School of Medicine, University of Pennsylvania, Philadelphia; 6 Renal-Electrolyte and Hypertension Division, Department of Medicine, Perelman School of Medicine at the University of Pennsylvania, Philadelphia; 7 Department of Public Health, University of Massachusetts-Lowell, Lowell; 8 Division of Cardiology, Columbia University Irving Medical Center, New York, New York; 9 Department of Preventive Medicine, University of Tennessee Health Science Center, Memphis; 10 Medical Service, Memphis VA Medical Center, Memphis, Tennessee; 11 Center for Healthful Behavior Change, Division of Health and Behavior, Department of Population Health, New York University School of Medicine, New York, New York

## Abstract

**Importance:**

Therapeutic inertia may contribute to racial and ethnic differences in blood pressure (BP) control.

**Objective:**

To determine the association between race and ethnicity and therapeutic inertia in the Systolic Blood Pressure Intervention Trial (SPRINT).

**Design, Setting, and Participants:**

This cross-sectional study was a secondary analysis of data from SPRINT, a randomized clinical trial comparing intensive (<120 mm Hg) vs standard (<140 mm Hg) systolic BP treatment goals. Participants were enrolled between November 8, 2010, and March 15, 2013, with a median follow-up 3.26 years. Participants included adults aged 50 years or older at high risk for cardiovascular disease but without diabetes, previous stroke, or heart failure. The present analysis was restricted to participant visits with measured BP above the target goal. Analyses for the present study were performed in from October 2020 through March 2021.

**Exposures:**

Self-reported race and ethnicity, mutually exclusively categorized into groups of Hispanic, non-Hispanic Black, or non-Hispanic White participants.

**Main Outcomes and Measures:**

Therapeutic inertia, defined as no antihypertensive medication intensification at each study visit where the BP was above target goal. The association between self-reported race and ethnicity and therapeutic inertia was estimated using generalized estimating equations and stratified by treatment group. Antihypertensive medication use was assessed with pill bottle inventories at each visit. Blood pressure was measured using an automated device.

**Results:**

A total of 8556 participants, including 4141 in the standard group (22 844 participant-visits; median age, 67.0 years [IQR, 61.0-76.0 years]; 1467 women [35.4%]) and 4415 in the intensive group (35 453 participant-visits; median age, 67.0 years [IQR, 61.0-76.0 years]; 1584 women [35.9%]) with at least 1 eligible study visit were included in the present analysis. Among non-Hispanic White, non-Hispanic Black, and Hispanic participants, the overall prevalence of therapeutic inertia in the standard vs intensive groups was 59.8% (95% CI, 58.9%-60.7%) vs 56.0% (95% CI, 55.2%-56.7%), 56.8% (95% CI, 54.4%-59.2%) vs 54.5% (95% CI, 52.4%-56.6%), and 59.7% (95% CI, 56.5%-63.0%) vs 51.0% (95% CI, 47.4%-54.5%), respectively. The adjusted odds ratios in the standard and intensive groups for therapeutic inertia associated with non-Hispanic Black vs non-Hispanic White participants were 0.85 (95% CI, 0.79-0.92) and 0.94 (95% CI, 0.88-1.01), respectively. The adjusted odds ratios for therapeutic inertia comparing Hispanic vs non-Hispanic White participants were 1.00 (95% CI, 0.90-1.13) and 0.89 (95% CI, 0.79-1.00) in the standard and intensive groups, respectively.

**Conclusions and Relevance:**

Among SPRINT participants above BP target goal, this cross-sectional study found that therapeutic inertia prevalence was similar or lower for non-Hispanic Black and Hispanic participants compared with non-Hispanic White participants. These findings suggest that a standardized approach to BP management, as used in SPRINT, may help ensure equitable care and could reduce the contribution of therapeutic inertia to disparities in hypertension.

**Trial Registration:**

ClinicalTrials.gov identifier: NCT01206062

## Key Points

**Question:**

Is the prevalence of therapeutic inertia for the treatment of hypertension similar across racial and ethnic participant groups?

**Findings:**

In this secondary cross-sectional analysis of 8556 participants in the Systolic Blood Pressure Intervention Trial , among participants with blood pressure above their randomized treatment goal, therapeutic inertia occurred at similar frequencies among non-Hispanic White and Hispanic participants but was lower among non-Hispanic Black vs non-Hispanic White participants.

**Meaning:**

These findings suggest that implementing highly standardized blood pressure measurement and treatment protocols in clinical practice, such as those imposed in clinical trial settings, may reduce racial and ethnic disparities in blood pressure control.

## Teaser

This cross-sectional study examines whether racial and ethnic differences were present for therapeutic inertia among participants in the Systolic Blood Pressure Intervention Trial.

## Introduction

Hypertension remains a leading modifiable cause of racial disparities in cardiovascular disease (CVD).^1,2^ Although blood pressure (BP) control rates improved from 1999 to 2010, they recently declined, especially among Hispanic and non-Hispanic Black adults with hypertension taking antihypertensive medication.^1,3^ Despite similar treatment rates and increased availability of safe, effective, and affordable antihypertensive medications, BP control rates among non-Hispanic Black and Hispanic adults remain significantly lower than for non-Hispanic White adults among US adults who report taking antihypertensive medication (53.2%, 58.2%, vs 68.2%, respectively).^3^ Additionally, non-Hispanic Black and Hispanic adults have a greater prevalence of comorbid diseases associated with pharmacologic treatment resistance, such as chronic kidney disease, obesity, and diabetes.^4^ Overall, 51.1% of non-Hispanic White individuals with hypertension in the US have a controlled BP (ie, systolic/diastolic BP of <140/90 mm Hg), compared with only 42.7% of Black and 42.4% of Hispanic individuals.^5^

The root causes of disparities in BP control are multifactorial and can be attributed to patient-related, clinician-related, and health care system–related barriers.^6,7,8,9^ Therapeutic inertia, the phenomenon of clinicians not initiating or up-titrating clinically indicated therapy in the setting of unmet treatment goals, is a clinician-related barrier to controlled BP.^6,7,10,11^ The prevalence of therapeutic inertia in hypertension in clinical practice remains high. However, data on racial and ethnic differences in therapeutic inertia in hypertension are limited, and the available data are conflicting. Some studies suggest that therapeutic inertia may be higher, while others show similar or lower prevalence, in Black vs White adults with hypertension.^12,13,14^ By studying racial and ethnic differences in hypertension-related therapeutic inertia in a clinical trial in which BP care was standardized and protocolized, targeted interventions could be developed and prioritized to improve BP management in all racial and ethnic groups, potentially leading to reduced disparities in BP control and hypertension-related CVD.

We tested whether racial and ethnic differences in therapeutic inertia in hypertension were present when BP care was standardized and protocolized. To do so, we used data from the Systolic Blood Pressure Intervention Trial (SPRINT), which randomized US adults with high CVD risk to intensive (ie, systolic BP [SBP] <120 mm Hg) vs standard treatment (ie, SBP <140 mm Hg) and used a standardized approach to both BP measurement and treatment. The SPRINT protocol required intensification of antihypertensive medication if SBP was not at goal. This design allowed for a quasinatural experimental comparison of therapeutic inertia by race and ethnicity in a randomized clinical trial in which BP management was standardized across racial and ethnic groups, including access, measurement, and treatment. Using SPRINT data, we sought to determine the association between self-reported race and ethnicity (ie, Hispanic, non-Hispanic Black, and non-Hispanic White) and therapeutic inertia in hypertension management.

## Methods

The current serial, cross-sectional, secondary analysis of SPRINT adheres to the Strengthening the Reporting of Observational Studies in Epidemiology (STROBE) guideline for reporting observational analyses.^15^ Each SPRINT site’s institutional review board approved the primary study protocol, and written informed consent was obtained from each participant. This secondary analysis was determined to be exempt by the University of Utah’s institutional review board because the project did not meet the definition for human participant research according to federal regulations.

The rationale, design, and primary results of SPRINT have been published.^16,17^ SPRINT was a multicenter, randomized trial that recruited adults with high CVD risk aged 50 years or older with SBP between 130 and 180 mm Hg depending on the number of antihypertensive medications being taken. Participants were enrolled between November 8, 2010, and March 15, 2013, and randomized to an intensive SBP goal of less than 120 mm Hg or a standard SBP goal of less than 140 mm Hg. Those with diabetes, prior history of stroke, heart failure, proteinuria of1 g/d of higher, or estimated glomerular filtration rate (eGFR) of less than 20 mL/min/1.73 m^2^ were excluded. Participants were followed monthly for the first 3 months and then every 3 months thereafter (median follow-up time 3.26 years). Additional study visits were available monthly for medication titration. Participating clinicians were encouraged to add an antihypertensive medication or up-titrate the dose until the SBP goal was achieved. To minimize the likelihood of therapeutic inertia, SPRINT mandated the use of milepost visits every 6 months in the intensive group, but not the standard group, at which time BP was measured, and a new medication was added if necessary.^18^ Because of this design element, we stratified all analyses by treatment group. Antihypertensive medications were provided free of charge at each participant visit with sufficient medication to last through the next follow-up visit without the need for pharmacy fills outside of the trial. We restricted the analysis to participant visits where SBP was above the randomized treatment goal within each treatment group. We excluded participant visits where SBP was at or below goal, as therapeutic inertia can only occur at participant visits where the SBP is above goal.

### Study Population

**Figure 1.**
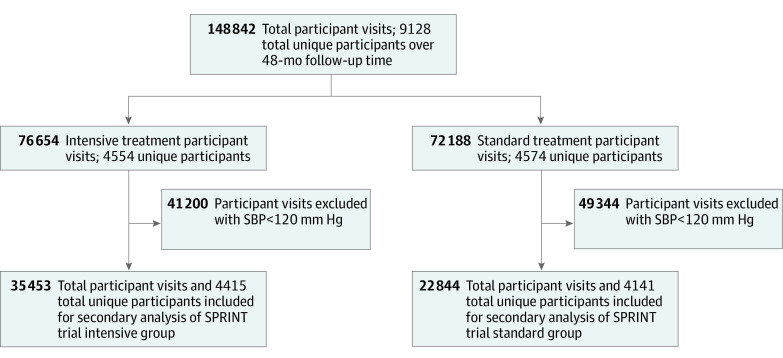
Flowchart Showing Eligibility Criteria for Inclusion in Current Study SBP indicates systolic blood pressure; SPRINT, Systolic Blood Pressure Intervention Trial.

There were 9361 participants randomized to either intensive treatment (4678 participants) or standard treatment (4683 participants). After restricting the sample to study visits where SBP was above target goal, there were 4141 and 4415 unique participants with at least 1 visit with measured SBP above the target goal, representing 22 844 and 35 453 participant-visits in the standard and intensive treatment groups, respectively (Figure 1).

### Definition of Self-reported Race and Ethnicity

Self-reported race and ethnicity, categorized as Hispanic, non-Hispanic Black, and non-Hispanic White, was the primary exposure for analysis. Participants were first identified by self-reported ethnicity, categorized as Hispanic or non-Hispanic. Then, participants were categorized by self-reported race, defined as Black or African American, White or Caucasian, Native American or Alaska Native, Asian, Native Hawaiian or other Pacific Islander, and other. In the current study, 984 participants were included as Hispanic irrespective of self-identified race. Participants who identified as non-Hispanic and Black or African American alone were included as non-Hispanic Black (n = 2802). Participants who identified solely as White were included as non-Hispanic White (n = 5399). We could not analyze Native American or Alaska Native, Asian, Native Hawaiian or other Pacific Islander owing to low sample size, as these groups represented less than 3% of the study sample.

### Definition of Therapeutic Inertia

The primary outcome, therapeutic inertia, was defined as no change or a reduction in the participant’s antihypertensive medication regimen intensity during a study visit when SBP was measured at or above the assigned target goal (ie, SBP ≥120 mm Hg for those in the intensive treatment group and SBP ≥140 mm Hg for those randomized to the standard treatment group). The unit of analysis for therapeutic inertia was each unique study visit. Change in the antihypertensive medication regimen intensity was quantified using the modified therapeutic intensity score (mTIS). Detailed description of how the mTIS was calculated in SPRINT is provided in the eMethods in the Supplement. The qualitative change (increase, decrease, or no change) in the mTIS at each visit was used to determine whether therapeutic inertia occurred at each study visit. Therapeutic inertia was considered to have occurred if the change in the mTIS at each visit was negative or zero (ie, per the mTIS, the medication regimen was de-escalated or not intensified when the SBP was at or above the assigned target goal). Therapeutic inertia did not occur if a participant had a positive change in the mTIS (ie, per the mTIS, the medication regimen was intensified when the SBP was at or above the assigned target goal).

### Other Study Measures

Participants’ sociodemographic data were collected at baseline. Clinical, laboratory, and medication data were collected at baseline and every 3 months thereafter by trained study personnel.^16,17,18^ Blood pressure was measured at baseline and each study visit using an automated device (Omron-HEM-907 XL; Omron Healthcare) following a standardized procedure by trained clinical staff.^19^ Because treatment-related serious adverse events (SAE) can influence therapeutic inertia, we included treatment-related SAEs of interest (orthostatic hypotension with symptoms, electrolyte problems, syncope, injurious fall, acute kidney injury, hypotension, and bradycardia) occurring in the 1 month before each visit in the current analysis as a covariate.^16,17,18^

### Statistical Analysis

All analyses were stratified by the treatment group (intensive vs standard) for 2 reasons. First, the intensive treatment group mandated milepost visits every 6 months to ensure that antihypertensive intensification was occurring in concordance with SPRINT protocol, whereas the standard treatment group did not have milepost visits.^18^ Second, the intensive treatment group had approximately 10% more total study visits than the standard treatment group. Analysis was restricted to the first 48 months of follow-up, as sample sizes decreased substantially thereafter.

Baseline characteristics were compared by self-reported race and ethnicity (Hispanic, non-Hispanic Black, and non-Hispanic White). *P *values were calculated for comparison of characteristics by race and ethnicity only (not therapeutic inertia). This calculation was done by *t *tests or Kruskal Wallis tests when appropriate for parametric or nonparametric continuous variables, and χ^2^ tests for comparison of categorical variables. Tests were all 2-seded with a *P *value cutoff of <.05. We determined the association between race and ethnicity and therapeutic inertia during SPRINT follow-up using a multivariable generalized estimating equation model to account for within-subject correlation. An adjusted odds ratio (OR) and 95% CI for therapeutic inertia associated with self-reported race and ethnicity was generated with non-Hispanic White as the reference group. Five nested models were created. Model 1 included race and ethnicity and follow-up time as the only fixed effects. Model 2 included variables in model 1 in addition to adjustment for sociodemographic variables and baseline values of clinical measurements (all variables listed in Table 1) and mTIS reported at the prior visit. Model 3 included sociodemographic variables in model 2 and follow-up clinical measurements, mTIS reported at the prior visit, and whether a treatment-related SAE occurred within 1 month before the study visit. Model 4 was adjusted with the same covariates as model 3, with the addition of the degree to which SBP was above the assigned target goal and the cumulative number of prior study visits with therapeutic inertia. Model 5 was adjusted with the same covariates as model 4, with the addition of an interaction term between race and ethnicity and time. We used the same models described above to determine participant factors associated with therapeutic inertia overall and within racial and ethnic groups. Last, we used marginal standardization methods to graph the average model-based multivariable-adjusted estimated probability of therapeutic inertia at each participant visit by race and ethnicity (ie, model 3) in the intensive and standard groups separately.^20^ Though SPRINT was designed to achieve an SBP goal of 135 to 139 mm Hg in the standard group, intensification was only indicated if SBP was at least 140 mm Hg in the standard group at 2 consecutive study visits or at least 160 mm Hg at a single visit. As such, we performed 2 sensitivity analyses for model 3. The first sensitivity analysis was performed after redefining therapeutic inertia to require 2 consecutive study visits where SBP was above goal with no change or a reduction in the participant’s antihypertensive medication regimen intensity for both randomized treatment groups. The second sensitivity analysis was restricted to the standard group and required either 1 study visit with SBP ≥160 mm Hg or 2 consecutive study visits with SBP ≥140 mm Hg. Analyses for the current report were performed using R, version 4.0.0 (R Foundation for Statistical Computing), from October 2020 through March 2021.

**Table 1.** Baseline Characteristics of Systolic Blood Pressure Intervention Trial Participants Included in the Current Analysis by Treatment Group and Race and Ethnicity

Variable	Treatment Group, No. (%)					
	Standard (n = 4141)			Intensive (n = 4415)		
	Non-Hispanic White (n = 2451)	Non-Hispanic Black (n = 1306)	Hispanic (n = 384)	Non-Hispanic White (n = 2639)	Non-Hispanic Black (n = 1329)	Hispanic (n = 447)
Age, median (IQR), y	**70 (63-77)**	**63 (57-70)^a^**	**64 (58-72)^a^**	**70 (64-77)**	**62 (57-71)^a^**	**64 (58-72)^a^**
Sex						
Male	1760 (72)	714 (55)	200 (52)	1861 (71)	721 (54)	249 (56)
Female	691 (28)	592 (45)^a^	184 (48)^a^	778 (29)	608 (46)^a^	198 (44)^a^
High school level of education or less	2007 (82)	846 (65)^a^	227 (59)^a^	2153 (82)	852 (64)^a^	267 (60)^a^
Full-time employment	513 (21)	275 (21)	89 (23)	538 (20)	279 (21)	116 (26)
Retired	1614 (66)	669 (51)^a^	200 (52)^a^	1766 (67)	682 (51)^a^	226 (51)^a^
Lives with others	1793 (73)	849 (65)^a^	295 (77)^a^	1938 (73)	853 (64)^a^	334 (75)^a^
Health insurance status						
Uninsured	155 (6)	235 (18)^a^	40 (10)^a^	142 (5)	255 (19)^a^	63 (14)^a^
Medicare or Medicaid	1580 (64)	632 (48)^a^	197 (51)^a^	1725 (65)	592 (45)^a^	224 (50)^a^
Private	1090 (44)	471 (36)^a^	175 (46)^a^	1237 (47)	486 (37)^a^	190 (43)^a^
VA	583(24)	217 (17)^a^	34 (9)^a^	642 (24)	224 (17)^a^	50 (11)^a^
Usual source of care						
No usual source	88 (4)	60 (5)^a^	17 (4)^a^	82 (3)	64 (5)^a^	24 (5)^a^
Hospital or clinic	2144 (88)	1028 (79)	340 (89)	2324 (88)	1037 (78)	388 (87)
Community health center	210 (9)	215 (17)	27 (7)	231 (9)	225 (17)	35 (8)
Smoking status						
Never	1007 (41)	577 (44)^a^	235 (61)^a^	1096 (42)	582 (44)^a^	246 (55)^a^
Current	217 (9)	286 (22)	33 (9)	234 (9)	312 (23)	64 (14)
Former	1227 (50)	443 (34)	116 (30)	1309 (50)	435 (33)	137 (31)
BMI, median (IQR)	29 (26-32)	30 (27-34)^a^	28 (26-31)^a^	29 (26-32)	30 (27-35)^a^	29 (26-33)^a^
SBP, median (IQR), mm Hg	139 (130-149)	139 (130-150)	141 (132-151)	139 (130-149)	138 (128-150)	140 (131-150)
eGFR, median (IQR), mL/min/1.73 m^2^	68 (56-79)	77 (62-91)^a^	77 (63-89)^a^	68 (56-80)	76 (61-91)^a^	77 (64-91)^a^
Serum potassium, median (IQR), mmol/L	4 (4-4)	4 (4-4)^a^	4 (4-4)^a^	4 (4-4)	4 (4-4)^a^	4 (4-4)^a^
Serum sodium, median (IQR), mEq/L	140 (139-142)	141 (139-142)^a^	140 (139-142)^a^	140 (139-142)	141 (139-142)^a^	141 (139-142)^a^
Depression	455 (19)	225 (17)	79 (21)	494 (19)	222 (17)	90 (20)
No. of antihypertensive medications, median (IQR)	2 (1-3)	2 (1-3)^a^	2 (1-2)^a^	2 (1-3)	2 (1-3)^a^	2 (1-3)^a^
mTIS, median (IQR)	1 (0-2)	1 (1-2)^a^	1 (0-2)^a^	1 (1-2)	2 (1-2)^a^	1 (1-2)^a^
ACEI or ARB	1468 (60)	686 (53)^a^	247 (64)^a^	1560 (59)	734 (55)^a^	297 (66)^a^
CCB	764 (31)	614 (47)^a^	130 (34)^a^	839 (32)	581 (44)^a^	136 (30)^a^
Thiazide diuretic	970 (40)	671(51)^a^	118 (31)^a^	944 (36)	642 (48)^a^	159 (36)^a^
Loop diuretic	132 (5)	78 (6)	16 (4)	153 (6)	92 (7)	15 (3)
Beta-blocker	924 (38)	424 (32)^a^	119 (31)^a^	1041 (39)	460 (35)^a^	153 (34)^a^
Alpha-blocker	272 (11)	101 (8)^a^	28 (7)^a^	310 (12)	108 (8)^a^	33 (7)^a^
No. of non-antihypertensive medications, median (IQR)	3 (1-6)	2 (1-5)^a^	2 (1-4)^a^	4 (2-6)	3 (1-5)^a^	2 (1-4)^a^
Current medication use						
Statin	1221 (50)	459 (35)^a^	153 (40)^a^	1288 (49)	420 (32)^a^	171 (38)^a^
Aspirin	1439 (59)	564 (43)^a^	150 (39)^a^	1622 (61)	567 (43)^a^	177 (40)^a^

Abbreviations: ACEI, angiotensin converting enzyme inhibitor; ARB, angiotensin II receptor blocker; BMI, body mass index (calculated as weight in kilograms divided by height in meters squared); CCB, calcium channel blocker; eGFR, estimated glomerular filtration rate; mTIS, modified therapeutic intensity score; SBP, systolic blood pressure; VA, Veterans Affairs.

^a^ Indicates statistically significant difference from non-Hispanic White participants (*P* < .05).

## Results

### Baseline Characteristics by Race and Ethnicity and Treatment Group

A total of 8556 participants were included in analyses. In the standard treatment group of 4141 participants (median age, 67.0 years [IQR, 61.0-76.0 years]; 1467 women [35.4%] and 2674 men [64.6%]), 384 were Hispanic (9.3%), 1305 were non-Hispanic Black (31.5%), and 2451 were non-Hispanic White (59.2%). In the intensive treatment group of 4415 participants (median age, 67.0 years [IQR, 61.0-76.0 years]; 1584 women [35.9%] and 2831 men [64.1%]), 447 were Hispanic (10.1%), 1329 were non-Hispanic Black (30.1%), and 2639 were non-Hispanic White (59.8%).

In both the standard and the intensive treatment groups, compared with non-Hispanic White participants, non-Hispanic Black and Hispanic participants were younger; had a higher proportion of participants who were female, uninsured, with no usual source of care, and never smokers; and had a lower proportion with a high school education or less, who were retired, and who were taking a statin or aspirin (Table 1). Missingness for baseline characteristics are shown in eTable 1 in the Supplement. The differences between the participants’ measured BP and their randomized BP goal at 12-, 24-, 36-, and 48-month follow-up, stratified by randomized treatment group and race and ethnicity, are shown in eFigures 1, 2, 3, and 4 in the Supplement.

### Race and Ethnicity and Therapeutic Inertia—Standard Treatment Group

**Figure 2.**
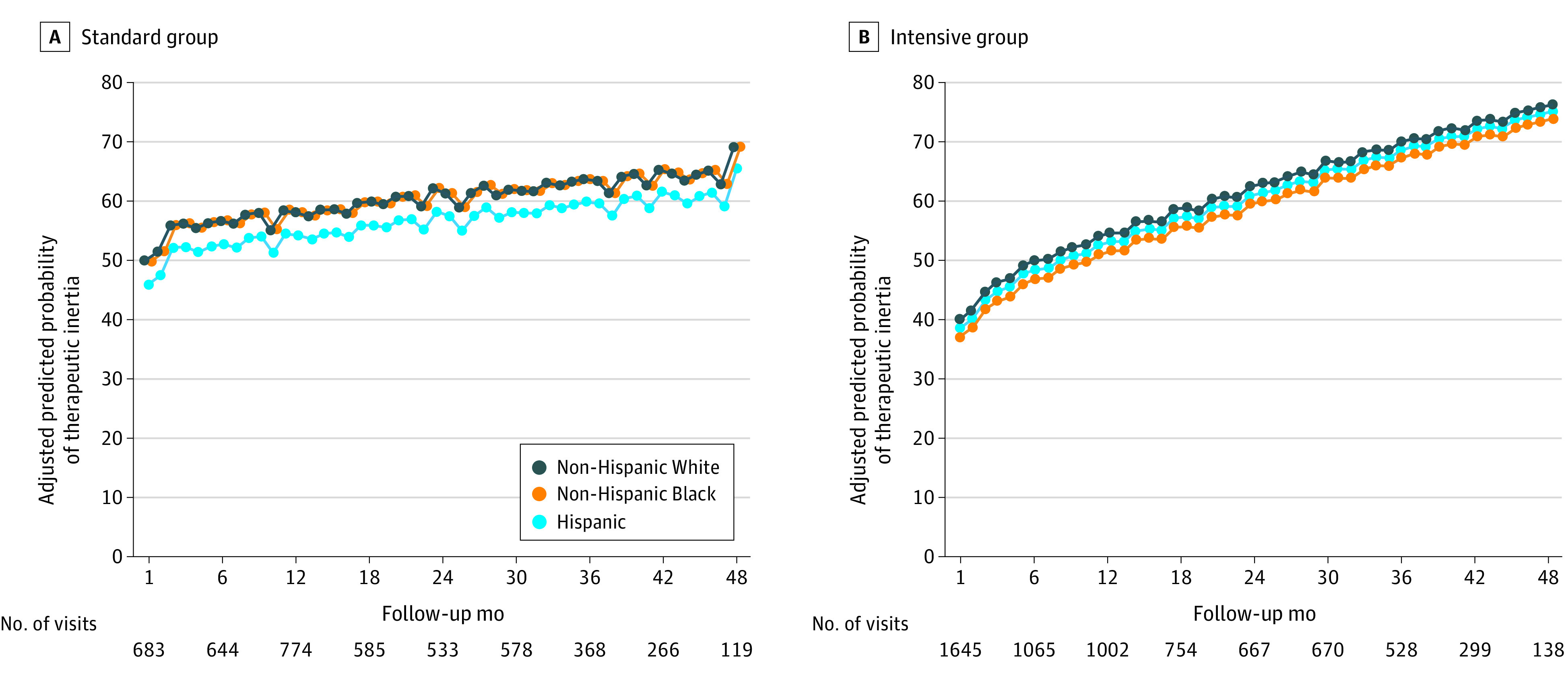
Predicted Prevalence of Therapeutic Inertia by Race and Ethnicity in the Standard and Intensive Treatment Arms of the Systolic Blood Pressure Intervention Trial Predicted probability of therapeutic inertia is shown for participants randomized to the standard treatment arm (A) and intensive treatment arm (B). This figure represents an average model-based, multivariable-adjusted estimated probability of therapeutic inertia at each participant visit by race and ethnicity averaged across the observed values of all other covariates in the logistic regression model. Prediction values are based on model 3, which is adjusted for race and ethnicity and time in addition to age, sex, education, employment, living with others, insurance status, source of care, smoking status, body mass index, depression, statin use, and aspirin use, as well as most recent visit SBP, eGFR, serum potassium, serum sodium, number of antihypertensive medications, prior mTIS, angiotensin converting enzyme inhibitor/angiotensin converting enzyme inhibitor, calcium channel blocker, thiazide diuretic, loop diuretic, beta-blocker, alpha-blocker, number of nonantihypertensive medications, and serious adverse events reported within 1 month before the study visit..

In the standard treatment group, the overall prevalence of therapeutic inertia was 59.8% (95% CI, 58.9%-60.7%), 56.8% (95% CI, 54.4%-59.2%), and 59.7% (95% CI, 56.6%-63.0%) among non-Hispanic White, non-Hispanic Black, and Hispanic participant-visits, respectively (Table 2). The adjusted predicted probability of therapeutic inertia increased over time (Figure 2A) in all racial and ethnic groups. The unadjusted prevalence of therapeutic inertia at 12 months was 55.7% (95% CI, 51.3%-60.1%), 54.3% (95% CI, 48.0%-60.4%), and 64.3% (95% CI, 51.2%-75.5%) for non-Hispanic White, non-Hispanic Black, and Hispanic participants, respectively, and was 61.9% (95% CI, 55.3%-68.1%), 54.0% (95% CI, 44.8%-62.9%), and 84.6% (95% CI, 70.3%-92.8%), at 36 months (Table 2).

**Table 2.** Odds Ratios for the Association Between Race and Ethnicity and Therapeutic Inertia in the Systolic Blood Pressure Intervention Trial by Randomized Treatment Arm

Variable	% (95% CI)		
	Non-Hispanic White	Non-Hispanic Black	Hispanic
**Standard arm**			
Unique participants, No.	2451	1306	384
Participant-visits, No.	13 726	7372	1746
Prevalence			
Overall^a^	59.8 (58.9-60.7)	56.8 (54.4-59.2)	59.7 (56.5-63.0)
12 Mo^b^	55.7 (51.3-60.1)	54.3 (48.0-60.4)	64.3 (51.2-75.5)
36 Mo^b^	61.9 (55.3-68.1)	54.0 (44.8-62.9)	84.6 (70.3-92.8)
Adjusted odds ratio (N = 4092)^b^^,^^c^^,^^d^	1 [Reference]	0.85 (0.79-0.92)	1.00 (0.90-1.13)
**Intensive arm**			
Unique participants, No.	2639	1329	447
Participant-visits, No.	22 319	10 716	2418
Prevalence			
Overall^a^	56.0 (55.2-56.7)	54.5 (52.4-56.6)	51.0 (47.4-54.5)
12 Mo^b^	54.8 (51.0-58.6)	51.4 (45.7-57.0)	35.5 (25.7-46.7)
36 Mo^b^	67.0 (62.0-71.7)	66.7 (58.9-73.6)	50.0 (33.6-66.4)
Adjusted odds ratio (N = 4377)^b^^,^^c^^,^^d^	1 [Reference]	0.94 (0.88-1.01)	0.89 (0.79-1.00)

^a^ This is the estimated overall prevalence of therapeutic inertia among included participant-visits across time (1 to 48 months) accounting for within-patient correlation.

^b^ This is the observed prevalence of therapeutic inertia among visits at 12 or 36 months.

^c^ Sample sizes for individual models represent total unique participants in the model.

^d^ Results are shown for Model 3 which is adjusted for race/ethnicity, time, age, sex, education, employment, living with others, insurance status, source of care, smoking status, body mass index, depression, statin use, aspirin use, as well as systolic blood pressure, eGFR, serum potassium, serum sodium, number of antihypertensive medications, prior mTIS, ACEI/ARB, CCB, thiazide diuretic, loop diuretic, beta-blocker, alpha-blocker, number of non-antihypertensive medications, plus adjustment for serious adverse events reported within 1 month prior of the study visit.

After adjustment for patient sociodemographic data, follow-up values of clinical measurements, mTIS reported at the prior visit, and treatment-related SAEs occurring within 1 month before the study visit (model 3), the OR for therapeutic inertia was 0.85 (95% CI, 0.79-0.92) comparing non-Hispanic Black vs non-Hispanic White participants (Table 2). When comparing Hispanic vs non-Hispanic White participants, the OR for therapeutic inertia was 1.00 (95% CI, 0.90-1.13). Progressive covariate adjustment in the nested models did not materially alter association estimates in the standard group (eTable 2 in the Supplement). In sensitivity analyses requiring 2 consecutive study visits with SBP of 140 mm Hg or greater without intensification, the OR for therapeutic inertia was lower in non-Hispanic Black participants (0.83 [95% CI, 0.73-0.94]) and Hispanic participants (0.73 [95% CI, 0.57-0.92]) compared with non-Hispanic White participants in the standard group (eTable 3 in the Supplement). Additionally, in sensitivity analysis following the standard group protocol for intensification requiring therapeutic inertia defined as either 1 visit with SBP of 160 mm Hg or greater or 2 consecutive visits with SBP of 140 mm Hg or greater, the OR for therapeutic inertia was 0.90 (95% CI, 0.81-1.02) in non-Hispanic Black participants and 0.82 (95% CI, 0.67-1.00) in Hispanic participants compared with non-Hispanic White participants (eTable 4 in the Supplement).

### Race and Ethnicity and Therapeutic Inertia—Intensive Treatment Group

The overall prevalence of therapeutic inertia across all included visits in the intensive group was 56.0% (95% CI, 55.2%-56,7%), 54.5% (95% CI, 52.4%-56.6%), and 51.0% (95% CI, 47.4%-54.5%) among non-Hispanic White, non-Hispanic Black, and Hispanic participant-visits, respectively. Compared with the standard group, the increase in the adjusted predicted probability of therapeutic inertia over time was greater in the intensive group (Figure 2B). At 12 months, the unadjusted prevalence of therapeutic inertia was 54.8% (95% CI, 51.0%-58.6%), 51.4% (95% CI, 45.7%-57.0%), and 35.5% (95% CI, 25.7%-46.7%) among non-Hispanic White, non-Hispanic Black, and Hispanic participants, respectively, which increased to 67.0% (95% CI, 62.0%-71.7%), 66.7% (95% CI, 58.9%-73.6%), and 50.0% (95% CI, 33.6%-66.4%) at 36 months (Table 2).

Comparing non-Hispanic Black vs non-Hispanic White participants, after adjustment for patient sociodemographic data, follow-up values of clinical measurements, mTIS reported at the prior visit, and treatment-related SAEs within 1 month prior to the study visit (model 3), the OR for therapeutic inertia was 0.94 (95% CI, 0.88-1.01) (Table 2). The multivariable-adjusted OR (model 3) for therapeutic inertia was 0.89 (95% CI, 0.79-1.00) comparing Hispanic and non-Hispanic White participants (Table 2). Progressive covariate adjustment did not materially alter association estimates in the intensive group (eTable 2 in the Supplement). In sensitivity analyses requiring 2 consecutive study visits with therapeutic inertia, the prevalence of therapeutic inertia was lower in Hispanic participants 0.78 (95% CI, 0.65-0.95) but not appreciably lower in non-Hispanic Black participants 0.93 (95% CI, 0.84-1.04) compared with non-Hispanic White participants in the intensive group (eTable 3 in the Supplement).

### Characteristics Associated With Therapeutic Inertia Overall in the Standard and Intensive Groups

In the multivariable-adjusted model (model 3) including all 3 racial and ethnic groups in the standard treatment group, follow-up time, older age, private insurance, higher eGFR, higher serum potassium, greater prior mTIS, calcium channel blocker use, and thiazide diuretic use were associated with a greater likelihood of therapeutic inertia (eTable 5 in the Supplement). Non-Hispanic Black race and ethnicity, full-time employment, higher body mass index, higher SBP, and angiotensin-converting enzyme inhibitor or angiotensin-II receptor blocker use were associated with a lower likelihood of therapeutic inertia.

In the multivariable-adjusted model (model 3) including all 3 racial and ethnic groups in the intensive treatment group, follow-up time, older age, greater prior mTIS, and thiazide diuretic use were associated with a greater likelihood of therapeutic inertia (eTable 6 in the Supplement). Higher body mass index, higher SBP, higher eGFR, angiotensin-converting enzyme inhibitor or angiotensin-II receptor blocker use, and alpha-blocker use were associated with a lower likelihood of therapeutic inertia.

### Characteristics Associated With Therapeutic Inertia by Race and Ethnicity

The majority of the characteristics had similar magnitude and direction of association with therapeutic inertia by racial and ethnic groups in both the standard and intensive treatment groups (eFigures 5 and 6 in the Supplement). However, age, female sex, full-time employment, private insurance, aspirin use, higher eGFR, higher serum potassium, thiazide diuretic use, number of nonantihypertensive medications, a treatment-related SAE reported within 1 month before the study visit, and number of prior visits with therapeutic inertia were differentially associated with therapeutic inertia by racial and ethnic groups in the standard treatment group (eFigure 5 in the Supplement), whereas age, Medicare or Medicaid insurance, greater body mass index, depression, aspirin use, higher SBP, prior mTIS, angiotensin-converting enzyme inhibitor or angiotensin-II receptor blocker use, calcium channel blocker use, thiazide diuretic use, and alpha-blocker use were differentially associated with therapeutic inertia by racial and ethnic groups in the intensive treatment group (eFigure 6 in the Supplement).

## Discussion

The Systolic Blood Pressure Intervention Trial used a standardized approach to BP measurement and treatment and had substantial representation of non-Hispanic Black and Hispanic participants. In this context, therapeutic inertia was either similar or lower for non-Hispanic Black and Hispanic participants compared with non-Hispanic White participants. This pattern was observed whether participants were randomized to the standard or intensive treatment group. These findings support the idea that a standardized approach to BP management as implemented in SPRINT may help ensure equitable care is provided to all patients and could reduce the contribution of therapeutic inertia to disparities in uncontrolled BP.

Despite similar awareness and treatment rates in the US, non-Hispanic Black and Hispanic adults continue to have substantially lower BP control rates compared with non-Hispanic White adults.^3,5^ It remains unclear to what extent differences in system- or clinician-level factors (eg, therapeutic inertia) vs patient-level factors (ie, antihypertensive medication nonadherence) contribute to this disparity. Medication adherence is often attributed as a driver of disparities in BP control. Separate analyses of patients with Medicare and private insurance suggest that nonadherence to antihypertensive medications is more common in non-Hispanic Black and Hispanic patients.^21,22,23,24,25^ However, this association is attenuated after adjustment for other socioeconomic variables and fails to entirely explain the disparities in BP control by race and ethnicity.^21,22,23,24,25^

Data on racial and ethnic differences in therapeutic inertia, defined as the act of not intensifying a medication regimen despite being indicated, is limited and conflicting. One multicenter cohort of 16 881 hypertensive adults showed therapeutic inertia to occur in 67.9% of clinic visits for Black patients and 72.2% in White patients.^12^ In a study of adults with hypertension on antihypertensive medication, treatment intensification with additional antihypertensive medication occurred in only 13.8%, 10.3%, and 10.5% of visits for non-Hispanic Black, Hispanic, and non-Hispanic White adults, respectively.^26^ When studying patients with hypertension from a safety-net hospital, Manze et al^13^ found higher rates of therapeutic inertia for Black patients. The current report suggests therapeutic inertia is either similar or lower in non-Hispanic Black and Hispanic adults compared with non-Hispanic White adults when care is standardized and protocolized as in SPRINT.

Therapeutic inertia has been identified as a key clinician-level barrier to BP control.^13,27,28,29^ A simulation by Bellows et al^30^ found that medication intensification was the most influential of the multiple interventions to achieve BP control. In community practice, therapeutic inertia is estimated to be present in more than 80% of clinic visits.^6,26^ In one analysis of managed care patients, clinicians increased antihypertensive medication in only 13% of visits where BP was uncontrolled.^31^ In the current analysis, therapeutic inertia was present in 50% to 60% of participant visits with uncontrolled BP. Although this rate is lower than estimates of therapeutic inertia in community practice, the prevalence was only slightly lower than the 60% to 70% reported in the Antihypertensive and Lipid-Lowering Treatment to Prevent Heart Attack Trial (ALLHAT).^32^ Although protocolization of care may be a significant driver of our findings, it is also possible that the overarching medical support available to participants of a clinical trial may have implications for addressing both therapeutic inertia and racial disparities in therapeutic inertia.

Prior research suggests that a standardized approach to BP management can increase BP control. For example, the American Medical Association’s Measure Accurately, Act Rapidly, and Partner with Patients BP Improvement Program reduced racial and ethnic disparities in BP control through addressing therapeutic inertia.^33,34^ Additionally, Kaiser Permanente Northern California has shown that a standardized approach to hypertension treatment can achieve parity in BP control between Black and White patients, with BP control exceeding 80% in both patient populations.^35^ Kaiser Permanente uses several strategies to reduce therapeutic inertia, including maintaining a system-wide hypertension registry, prioritizing evidence-based practice guidelines and fixed-dose combination therapy, and using medical assistants for follow-up measurements.^36,37^ While the SPRINT protocol for BP management represents one approach to standardizing BP care, other standardized approaches, such as the Measure Accurately, Act Rapidly, and Partner with Patients program and the Kaiser Permanente algorithm, demonstrate multiple effective strategies to minimize therapeutic inertia.^33,35,36,37^ In addition to implementing care protocols to reduce therapeutic inertia, health systems should encourage the use of fixed-dose combination medication (especially early in disease management), follow-up every 2 to 4 weeks after initiation or titration of drug therapy until BP goal is achieved, team-based care, and personalized feedback to clinicians.^38^ Greater implementation of patient-centered strategies are needed in community care of adults with hypertension.^39,40^ Ongoing studies focused on team-based hypertension care, mobile health platforms, and home BP monitoring will provide more evidence on interventions that increase patient engagement and reduce therapeutic inertia.^38^

In SPRINT, BP measurement and antihypertensive medication intensification were protocolized. This approach differs from usual community practice in which competing demands, time constraints, and clinical uncertainty in BP measurement can impact appropriate care delivery.^8^ By mandating antihypertensive medication intensification when BP was above target in SPRINT, inappropriate inaction (ie, clinician-level deviation from guideline recommendations or patient aversion to medication) was reduced, though not eliminated. For example, the current analysis adjusted for prior mTIS and the number of antihypertensive medications being taken, which were associated with greater therapeutic inertia. Clinicians might have been less likely to intensify an antihypertensive regimen if a patient was already taking a high dose or multiple antihypertensive medications. In contrast, the influence of appropriate inaction (ie, lifestyle modifications, contraindications to medication, or new comorbid conditions) on antihypertensive medication underuse is difficult to ascertain. All participants in SPRINT received education and counseling regarding physical activity, diet, smoking cessation, and weight management in order to minimize the effect that lifestyle modification may have on therapeutic inertia. Additionally, treatment-related SAEs were not associated with therapeutic inertia in either treatment group in the current analysis.

### Strengths and Limitations

There are several strengths and limitations of the current analysis. The Systolic Blood Pressure Intervention Trial was a large, racially and ethnically diverse, randomized clinical trial with high-quality BP measurement and detailed medication use including dosing levels at least every 3 months. Furthermore, the SPRINT treatment protocol achieved a large difference in SBP between treatment groups for the duration of the trial. The design of SPRINT allows for observation of therapeutic inertia in an environment where access, cost, and the approach to BP measurement and treatment are equal and standardized for all trial participants. However, as with all observational studies, there may be residual confounding. Unlike the intensive group, the standard group only indicated intensification if a participant was above SBP goal at 2 consecutive visits. Although the difference in protocol may translate into different intensification patterns between the standard and intensive groups, this limitation is addressed in the current study by stratifying the analysis by treatment group and the included sensitivity analyses defining therapeutic inertia as 2 consecutive visits without intensification for both treatment groups and as an SBP of 160 mm Hg or great or 2 consecutive study visits with an SBP of 140 mm Hg or greater in the standard group only. The current analysis did not evaluate the association between therapeutic inertia and BP control or antihypertensive intensification at subsequent study visits. Additionally, all interactions existed within the framework of a randomized clinical trial, limiting generalizability to community-treated patients in routine care. Finally, the SPRINT protocol mandated the use of milepost visits in the intensive group only, limiting direct comparisons of therapeutic inertia between study groups.

## Conclusions

In this cross-sectional study using data from SPRINT, therapeutic inertia was either similar or lower among non-Hispanic Black and Hispanic participants compared with non-Hispanic White participants. Therapeutic inertia represents a significant barrier to BP control, particularly among racial and ethnic minority populations. The findings of the current analysis indicate that well-resourced, protocolized, and standardized BP management—including BP measurement and medication initiation and intensification—may be an effective strategy to address racial and ethnic disparities in BP control driven by therapeutic inertia.

## Notes

Supplement. **eMethods.** Supplemental methods **eTable 1.** Missing baseline characteristics of SPRINT participants included in the current analysis by treatment arm **eTable 2.** Odds ratios for the association between race/ethnicity and therapeutic inertia in SPRINT by randomized treatment arm **eTable 3.** Odds ratios for the association between race/ethnicity and therapeutic inertia in SPRINT by randomized treatment arm in sensitivity analyses requiring two consecutive visits with therapeutic inertia **eTable 4.** Odds ratios for the association between race/ethnicity and therapeutic inertia in SPRINT in sensitivity analysis requiring two consecutive visits with an SBP > 140 mm Hg or a single visit with an SBP > 160 mm Hg in the standard arm **eTable 5.** Factors associated with therapeutic inertia among SPRINT participants in the standard arm **eTable 6.** Factors associated with therapeutic inertia among SPRINT participants in the intensive treatment arm **eFigure 1.** Boxplots and histograms of mm Hg over BP goal stratified by study arm and race/ethnicity at 12 months **eFigure 2.** Boxplots and histograms of mm Hg over BP goal stratified by study arm and race/ethnicity at 24 months **eFigure 3.** Boxplots and histograms of mm Hg over BP goal stratified by study arm and race/ethnicity at 36 months **eFigure 4.** Boxplots and histograms of mm Hg over BP goal stratified by study arm and race at 48 months **eFigure 5.** Factors associated with therapeutic inertia among SPRINT participants by race/ethnicity in the standard treatment arm **eFigure 6.** Factors associated with therapeutic inertia among SPRINT participants by race/ethnicity in the intensive treatment arm
eReferences
