# Correction in Article Information

**DOI:** 10.1001/jamanetworkopen.2022.0244

**Published:** 2022-02-04

In the Original Investigation titled “Analysis of Therapeutic Inertia and Race and Ethnicity in the Systolic Blood Pressure Intervention Trial: A Secondary Analysis of a Randomized Clinical Trial,”^1^ published January 10, 2022, a disclaimer was missing to note that author Olugbenga Ogedegbe is an Associate Editor of *JAMA Network Open*. This article has been corrected.^1^
