# Supplementary material for: Analysis of Therapeutic Inertia and Race and Ethnicity in the Systolic Blood Pressure Intervention Trial: A Secondary Analysis of a Randomized Clinical Trial
Source: JAMA Netw Open. 2022 Jan 10;5(1):e2143001. doi: 10.1001/jamanetworkopen.2021.43001 (PMC8749480; doi:10.1001/jamanetworkopen.2021.43001)
Supplement: Supplement. — eMethods. Supplemental methods eTable 1. Missing baseline characteristics of SPRINT participants included in the current analysis by treatment arm eTable 2. Odds ratios for the association between race/ethnicity and therapeutic inertia in SPRINT by randomized treatment arm eTable 3. Odds ratios for the association between race/ethnicity and therapeutic inertia in SPRINT by randomized treatment arm in sensitivity analyses requiring two consecutive visits with therapeutic inertia eTable 4. Odds ratios for the association between race/ethnicity and therapeutic inertia in SPRINT in sensitivity analysis requiring two consecutive visits with an SBP > 140 mm Hg or a single visit with an SBP > 160 mm Hg in the standard arm eTable 5. Factors associated with therapeutic inertia among SPRINT participants in the standard arm eTable 6. Factors associated with therapeutic inertia among SPRINT participants in the intensive treatment arm eFigure 1. Boxplots and histograms of mm Hg over BP goal stratified by study arm and race/ethnicity at 12 months eFigure 2. Boxplots and histograms of mm Hg over BP goal stratified by study arm and race/ethnicity at 24 months eFigure 3. Boxplots and histograms of mm Hg over BP goal stratified by study arm and race/ethnicity at 36 months eFigure 4. Boxplots and histograms of mm Hg over BP goal stratified by study arm and race at 48 months eFigure 5. Factors associated with therapeutic inertia among SPRINT participants by race/ethnicity in the standard treatment arm eFigure 6. Factors associated with therapeutic inertia among SPRINT participants by race/ethnicity in the intensive treatment arm eReferences [file jamanetwopen-e2143001-s001.pdf]

Supplemental Online Content

Zheutlin AR, Mondesir FL, Derington CG, et al. Analysis of therapeutic inertia and race and ethnicity in the systolic blood pressure intervention trial: a secondary analysis of a randomized clinical trial. *JAMA Netw Open*. 2022;5(1):e2143001. doi:10.1001/jamanetworkopen.2021.43001

eMethods. Supplemental methods

eTable 1. Missing baseline characteristics of SPRINT participants included in the current analysis by treatment arm

eTable 2. Odds ratios for the association between race/ethnicity and therapeutic inertia in SPRINT by randomized treatment arm

eTable 3. Odds ratios for the association between race/ethnicity and therapeutic inertia in SPRINT by randomized treatment arm in sensitivity analyses requiring two consecutive visits with therapeutic inertia

eTable 4. Odds ratios for the association between race/ethnicity and therapeutic inertia in SPRINT in sensitivity analysis requiring two consecutive visits with an SBP > 140 mm Hg or a single visit with an SBP > 160 mm Hg in the standard arm

eTable 5. Factors associated with therapeutic inertia among SPRINT participants in the standard arm

eTable 6. Factors associated with therapeutic inertia among SPRINT participants in the intensive treatment arm

eFigure 1. Boxplots and histograms of mm Hg over BP goal stratified by study arm and race/ethnicity at 12 months

eFigure 2. Boxplots and histograms of mm Hg over BP goal stratified by study arm and race/ethnicity at 24 months

eFigure 3. Boxplots and histograms of mm Hg over BP goal stratified by study arm and race/ethnicity at 36 months

eFigure 4. Boxplots and histograms of mm Hg over BP goal stratified by study arm and race at 48 months

eFigure 5. Factors associated with therapeutic inertia among SPRINT participants by race/ethnicity in the standard treatment arm

eFigure 6. Factors associated with therapeutic inertia among SPRINT participants by race/ethnicity in the intensive treatment arm

eReferences

This supplemental material has been provided by the authors to give readers additional information about their work.

© 2022 Zheutlin AR et al. *JAMA Network Open.*

eMethods. Supplemental Methods

In SPRINT, investigators' decisions to intensify therapy by increasing a medication dose or adding another antihypertensive medication was individualized per participant. To capture the intensity of medication regimens, we calculated a therapeutic intensity score (TIS) for each participant's antihypertensive regimen recorded at each included visit. The TIS is a validated summary measure that accounts for (1) the number of antihypertensive medications in a participant's regimen; and (2) the dose of each medication that the participant is receiving relative to the Food and Drug Administration (FDA) maximally-recommended dose.[1,2]

For the present analysis, the therapeutic intensity score (TIS) TIS was modified to use the 2017 American College of Cardiology/American Heart Association BP Guideline-defined maximum dose for each agent.[3] The mTIS is defined below, where n is the number of antihypertensive medications in the regimen and *i* is each individual antihypertensive medication.

$$mTIS = \sum_{i-1}^{n} \frac{Prescribed\ Daily\ Dose_i}{Guideline - Defined\ Maximum\ Daily\ Dose_i}$$

For example, the mTIS for a participant who is taking lisinopril 20 mg daily (maximum daily dose 40 mg) and metoprolol tartrate 25 mg twice-daily (maximum daily dose 200 mg) would be calculated as:

$$mTIS = \frac{lisinopril\ 20\ mg}{lisinopril\ 40\ mg} + \frac{metoprolol\ 50\ mg}{metoprolol\ 200\ mg} = \frac{1}{2} + \frac{1}{4} = 0.75$$

© 2021 Zheutlin AR et al. *JAMA Network Open.*

eTable 1. Missing baseline characteristics of SPRINT participants included in the current analysis by treatment arm

Variable	Standard Arm (N=4141)	Intensive Arm (N=4415)
	**Missing**	**Missing**
**Age –** Median (IQR)	0 (0)	0 (0)
**Female sex, n(%)**	0 (0)	0 (0)
**High school level of education or less, n(%)**	1 (0)	0 (0)
**Full-time employment, n(%)**	3 (0.1)	3 (0.1)
**Retired, n(%)**	1 (0)	0 (0)
**Lives with others, n(%)**	3 (0.1)	1 (0)
**Health insurance status, n(%)**		
Uninsured	1 (0)	0 (0)
Medicare/Medicaid	1 (0)	0 (0)
Private	1 (0)	0 (0)
VA	1 (0)	0 (0)
**Usual source of care, n(%)**		
No usual source	12 (0.3)	5 (0.1)
Hospital/clinic	-	-
Community health center	-	-
**Smoking status, n(%)**		
Never	0 (0)	0 (0)
Current	-	-
Former	-	-
**BMI** – Median (IQR)	32 (0.8)	23 (0.5)
**Depression, n(%)**	4 (0.1)	5 (0.1)
**Current medication use, n(%)**		
Statin	0 (0)	0 (0)
Aspirin	0 (0)	0 (0)
**SBP** – Median (IQR)	1 (0)	0 (0)
**eGFR** – Median (IQR)	23 (0.6)	15 (0.3)
**Serum potassium, mmol/L** – Median (IQR)	3 (0.1)	6 (0.1)
**Serum sodium, mEq/L** – Median (IQR)	3 (0.1)	6 (0.1)
**Number of antihypertensive medications** – Median (IQR)	0 (0)	0 (0)
**mTIS** – Median (IQR)	1 (0)	0 (0)
**ACEI or ARB, n(%)**	0 (0)	0 (0)
**CCB, n(%)**	0 (0)	0 (0)
**Thiazide diuretic, n(%)**	0 (0)	0 (0)
**Loop diuretic, n(%)**	0 (0)	0 (0)
**Beta-blocker, n(%)**	0 (0)	0 (0)
**Alpha-blocker, n(%)**	0 (0)	0 (0)
**Number of non-antihypertensive medications** – Median (IQR)	0 (0)	0 (0)

ACEI: angiotensin converting enzyme inhibitor; ARB: angiotensin II receptor blocker; BMI: body mass index; CCB: calcium channel blocker; eGFR: estimated glomerular filtration rate; IQR: interquartile range; mTIS: modified therapeutic intensity score; SBP: systolic blood pressure; SPRINT: Systolic Blood Pressure Intervention Trial; VA: Veterans Affairs.
There were 4,141 and 4,415 participants (22,844 and 35,453 participant-visits) who had a study visit where SBP was above target in the standard and intensive arms, respectively.

© 2022 Zheutlin AR et al. *JAMA Network Open.*

eTable 2. Odds ratios for the association between race/ethnicity and therapeutic inertia in SPRINT by randomized treatment arm.

	Standard arm		
	**Non-Hispanic White**	**Non-Hispanic Black**	**Hispanic**
**Odds ratio (95% CI)**			
Model 1 *N=4,141*†	1 (Reference)	0.89 (0.84, 0.95)	1.00 (0.91,1.11)
Model 2 *N=4,069*†	1 (Reference)	0.92 (0.86, 0.99)	1.01 (0.90,1.12)
Model 3 *N=4,092*†	1 (Reference)	0.85 (0.79, 0.92)	1.00 (0.90,1.13)
Model 4 *N=4092*†	1 (Reference)	0.88 (0.82,0.96)	1.08 (0.97,1.22)
Model 5 * *N=4,092*†	1 (Reference)	0.89 (0.82,0.97)	0.98 (0.86,1.13)
	**Intensive arm**		
	**Non-Hispanic White**	**Non-Hispanic Black**	**Hispanic**
**Odds ratio (95% CI)**	-	-	-
Model 1 *N=4,415*†	1 (Reference)	0.94 (0.90, 1.00)	0.86 (0.76, 0.95)
Model 2 *N=4,364*†	1 (Reference)	0.96 (0.90, 1.02)	0.87 (0.78, 0.97)
Model 3 *N=4,377*†	1 (Reference)	0.94 (0.88, 1.01)	0.89 (0.79, 1.00)
Model 4 *N=4377*†	1 (Reference)	0.99 (0.92,1.05)	0.99 (0.87,1.10)
Model 5 * *N=4,377*†	1 (Reference)	0.99 (0.92,1.05)	0.95 (0.84,1.06)

† Sample sizes for individual models represent total unique participants in the model.
*Due to the interaction between race/ethnicity and month, odds ratio changes over time. Reported here is the estimated odds ratio at month 12
Model 1 included race/ethnicity and time as the only fixed effects.
Model 2 was adjusted for race and time, in addition to age, sex, education, employment, living with others, insurance status, source of care, smoking status, BMI, depression, statin use, aspirin use, as well as baseline SBP, eGFR, serum potassium, serum sodium, number of antihypertensive medications, prior mTIS, ACEI/ARB, CCB, thiazide diuretic, loop diuretic, beta-blocker, alpha-blocker, and number of non-antihypertensive medications.
Model 3 included variables in Model 2 plus adjustment for follow-up clinical measurements and serious adverse events reported within one month prior of the study visit.
Model 4 included variables in Model 3 plus adjustment for the mm Hg the systolic blood pressure is above the treatment goal and the number of prior study visits with therapeutic inertia.
Model 5 included the same predictors as Model 4 except for the addition of an interaction between race/ethnicity and time.
CI: confidence interval; SPRINT: Systolic Blood Pressure Intervention Trial

© 2021 Zheutlin AR et al. *JAMA Network Open.*

eTable 3. Odds ratios for the association between race/ethnicity and therapeutic inertia in SPRINT by randomized treatment arm in sensitivity analyses requiring two consecutive visits with therapeutic inertia.

	Standard arm		
	**Non-Hispanic White**	**Non-Hispanic Black**	**Hispanic**
**Unique participants, n**	2451	1306	383
**Participant-visits, n**	13704	7364	1739
**Overall Prevalence, %* (95% CI)**	12.7 (12.0,13.5)	10.6 (9.0,12.4)	9.3 (7.1,11.9)
**12 Month Prevalence, %† (95% CI)**	10.8 (8.3,13.9)	10.5 (7.3,15.0)	1.8 (0.1,9.4)
**36 Month Prevalence, %† (95% CI)**	10.1 (6.8,14.8)	5.3 (2.5,11.1)	20.5 (10.8,35.5)
**§ Adjusted Odds ratio (95% CI) *N=4091*†‡**	1 (Reference)	0.83 (0.73,0.94)	0.73 (0.57,0.92)
	**Intensive arm**		
	**Non-Hispanic White**	**Non-Hispanic Black**	**Hispanic**
**Unique participants, n**	2638	1328	445
**Participant-visits, n**	22290	10688	2404
**Overall Prevalence, %* (95% CI)**	21.2 (20.4,22.1)	19.5 (17.3,21.7)	16.3 (13.3,20.1)
**12 Month Prevalence, %† (95% CI)**	20.3 (17.4,23.6)	17.8 (13.8,22.6)	10.5 (5.4,19.4)
**36 Month Prevalence, %† (95% CI)**	23.9 (19.8,28.6)	24.3 (18.2,31.7)	12.5 (5.0,28.1)
**§ Adjusted Odds ratio (95% CI) *N=4373***	ref	0.93 (0.84,1.04)	0.78 (0.65,0.95)

*This is the estimated overall prevalence of therapeutic inertia among included participant-visits across time (1 to 48 months) accounting for within-patient correlation.
†This is the observed prevalence of therapeutic inertia among visits at 12 or 36 months.
‡ Sample sizes for individual models represent total unique participants in the model.
§ Results are shown for Model 3 which is adjusted for race/ethnicity, time, age, sex, education, employment, living with others, insurance status, source of care, smoking status, BMI, depression, statin use, aspirin use, as well as SBP, eGFR, serum potassium, serum sodium, number of antihypertensive medications, prior mTIS, ACEI/ARB, CCB, thiazide diuretic, loop diuretic, beta-blocker, alpha-blocker, number of non-antihypertensive medications, plus adjustment for serious adverse events reported within one month prior of the study visit.
CI: confidence interval; SPRINT: Systolic Blood Pressure Intervention Trial

© 2021 Zheutlin AR et al. *JAMA Network Open.*

eTable 4. Odds ratios for the association between race/ethnicity and therapeutic inertia in SPRINT in sensitivity analysis requiring two consecutive visits with an SBP ≥ 140 mm Hg or a single visit with an SBP ≥ 160 mm Hg in the standard arm.

	Standard arm		
	**Non-Hispanic White**	**Non-Hispanic Black**	**Hispanic**
**Unique participants, n**	2451	1306	383
**Participant-visits, n**	13706	7364	1739
**Overall Prevalence, %[*] (95% CI)**	15.2 (14.3,16.0)	13.6 (11.7,15.6)	12.4 (9.9,15.4)
**12 Month Prevalence, %[†] (95% CI)**	12.7 (10.0,16.0)	13.4 (9.7,18.2)	1.8 (0.1,9.4)
**36 Month Prevalence, %[†] (95% CI)**	12.8 (9.0,17.9)	10.6 (6.2,17.6)	23.1 (12.6,38.3)
**§ Adjusted Odds ratio (95% CI)** ***N=4091[†‡]***	-	0.90 (0.81,1.02)	0.82 (0.67,1.00)

[*]This is the estimated overall prevalence of therapeutic inertia among included participant-visits across time (1 to 48 months) accounting for within-patient correlation.
[†]This is the observed prevalence of therapeutic inertia among visits at 12 or 36 months.
‡ Sample sizes for individual models represent total unique participants in the model.
§ Results are shown for Model 3 which is adjusted for race/ethnicity, time, age, sex, education, employment, living with others, insurance status, source of care, smoking status, BMI, depression, statin use, aspirin use, as well as SBP, eGFR, serum potassium, serum sodium, number of antihypertensive medications, prior mTIS, ACEI/ARB, CCB, thiazide diuretic, loop diuretic, beta-blocker, alpha-blocker, number of non-antihypertensive medications, plus adjustment for serious adverse events reported within one month prior of the study visit.
CI: confidence interval; SPRINT: Systolic Blood Pressure Intervention Trial

© 2021 Zheutlin AR et al. *JAMA Network Open.*

eTable 5. Factors associated with therapeutic inertia among SPRINT participants in the standard arm

	Odds Ratio (95% CI)				
**Variable**	**Model 1**	**Model 2**	**Model 3**	**Model 4**	**Model 5**
**Non-Hispanic White**	**1 (Reference)**	**1 (Reference)**	**1 (Reference)**	**1 (Reference)**	**1 (Reference)**
**Non-Hispanic Black**	0.89 (0.84,0.95)	0.92 (0.86, 0.99)	0.85 (0.79, 0.92)	0.88 (0.82,0.96)	0.89 (0.82,0.97)
**Hispanic**	1.00 (0.91,1.11)	1.01 (0.90, 1.12)	1.00 (0.90, 1.13)	1.08 (0.97,1.22)	0.98 (0.86,1.13)
**Follow-up time** – per one year increase	1.14 (1.11,1.17)	1.14 (1.11, 1.17)	1.15 (1.12, 1.18)	1.13 (1.08,1.16)	1.12 (1.06,1.17)
**Age –** per 10 year increase	-	1.06 (1.02, 1.11)	1.09 (1.04, 1.15)	1.14 (1.08,1.20)	1.14 (1.07,1.20)
**Female sex**	-	0.99 (0.93, 1.06)	1.00 (0.93, 1.08)	1.08 (1.00,1.16)	1.08 (1.00,1.16)
**Education**					
High school graduate or lower	**1 (Reference)**	**1 (Reference)**	**1 (Reference)**	**1 (Reference)**	**1 (Reference)**
Post High school training/education	-	1.00 (0.93, 1.07)	1.00 (0.93, 1.08)	0.98 (0.90,1.06)	0.98 (0.90,1.06)
**Full-time employment**	-	0.88 (0.79, 0.97)	0.87 (0.78, 0.96)	0.84 (0.74,0.93)	0.84 (0.74,0.93)
**Retired**	-	0.92 (0.84, 1.02)	0.91 (0.83, 1.00)	0.90 (0.81,1.00)	0.90 (0.81,1.00)
**Lives with others**	-	1.06 (0.98, 1.13)	1.05 (0.98, 1.13)	1.06 (0.98,1.14)	1.06 (0.98,1.14)
**Insurance status**					
Uninsured	**1 (Reference)**	**1 (Reference)**	**1 (Reference)**	**1 (Reference)**	**1 (Reference)**
Medicare/Medicaid	-	1.05 (0.96, 1.13)	1.07 (0.98, 1.18)	1.05 (0.96,1.16)	1.05 (0.96,1.17)
Private	-	1.11 (1.04, 1.19)	1.10 (1.02, 1.18)	1.08 (1.00,1.17)	1.08 (1.00,1.17)
VA	-	1.05 (0.96, 1.14)	1.05 (0.96, 1.15)	1.04 (0.94,1.15)	1.04 (0.94,1.15)
**Source of care**					
No usual source	**1 (Reference)**	**1 (Reference)**	**1 (Reference)**	**1 (Reference)**	**1 (Reference)**
Hospital/clinic	-	1.07 (0.90, 1.27)	1.00 (0.82, 1.22)	1.01 (0.82,1.21)	1.01 (0.83,1.22)
Community health center	-	1.14 (0.96, 1.39)	1.04 (0.86, 1.30)	1.05 (0.85,1.30)	1.06 (0.85,1.30)
**Smoking status**					
Never	**1 (Reference)**	**1 (Reference)**	**1 (Reference)**	**1 (Reference)**	**1 (Reference)**
Current	-	0.94 (0.85, 1.03)	0.92 (0.83, 1.02)	0.96 (0.87,1.07)	0.96 (0.87,1.07)
Former	-	1.01 (0.95, 1.08)	1.01 (0.94, 1.08)	1.02 (0.94,1.09)	1.01 (0.94,1.08)
**BMI, kg/m^2 –** per 5 kg/m² increase	-	0.97 (0.94, 1.00)	0.94 (0.91, 0.97)	0.93 (0.90,0.95)	0.92 (0.90,0.95)
**SBP, mm Hg –** per 10 mm Hg increase	-	0.99 (0.97, 1.01)	0.71 (0.69, 0.73)	0.75 (0.73,0.77)	0.75 (0.73,0.77)
**eGFR, mL/min/1.73m^2** – per 10 mL/min/1.73m2 increase	-	1.01 (1.00, 1.03)	1.03 (1.02, 1.05)	1.03 (1.00,1.04)	1.03 (1.01,1.04)
**Serum potassium, mmol/L –** per 0.5 mmol/L increase	-	1.05 (0.98, 1.13)	1.19 (1.10, 1.27)	1.18 (1.09,1.27)	1.18 (1.09,1.27)
**Serum sodium, mEq/L –** per 5 mEq/L increase	-	1.09 (0.97, 1.22)	1.09 (0.98, 1.22)	1.04 (0.92,1.18)	1.04 (0.92,1.18)
**Depression**	-	0.98 (0.90, 1.05)	0.98 (0.90, 1.07)	1.00 (0.92,1.10)	1.00 (0.92,1.09)
**Number of antihypertensive medications** – per medication increase	-	0.93 (0.86, 1.01)	1.08 (0.96, 1.21)	1.10 (0.95,1.25)	1.10 (0.96,1.25)
**Prior mTIS**	-	1.05 (1.01, 1.08)	1.21 (1.13, 1.28)	1.25 (1.17,1.34)	1.25 (1.17,1.34)
**ACEI or ARB**	-	0.94 (0.86, 1.04)	0.82 (0.73, 0.93)	0.83 (0.72,0.95)	0.83 (0.72,0.95)
**CCB**	-	1.09 (0.99, 1.22)	1.17 (1.03, 1.33)	1.06 (0.92,1.21)	1.06 (0.92,1.21)
**Thiazide diuretic**	-	1.05 (0.94, 1.17)	1.24 (1.09, 1.40)	1.18 (1.03,1.35)	1.18 (1.04,1.35)
**Loop diuretic**	-	1.07 (0.91, 1.25)	1.05 (0.87, 1.24)	1.05 (0.87,1.28)	1.06 (0.87,1.29)
**Beta-blocker**	-	1.10 (1.00, 1.22)	0.93 (0.82, 1.07)	0.97 (0.83,1.12)	0.97 (0.83,1.12)
**Alpha-blocker**	-	1.03 (0.91, 1.16)	0.83 (0.70, 1.00)	0.80 (0.67,0.97)	0.81 (0.67,0.98)
**Number of non-antihypertensive medications** – per medication increase	-	0.99 (0.98, 1.00)	0.99 (0.98, 1.00)	0.99 (0.97,1.00)	0.99 (0.97,1.00)
**Current medication**					
Statin	-	1.01 (0.95, 1.08)	1.00 (0.94, 1.07)	0.99 (0.92,1.06)	0.99 (0.92,1.06)
Aspirin	-	0.98 (0.92, 1.04)	0.94 (0.88, 1.00)	0.92 (0.86,0.98)	0.93 (0.87,0.99)
Collected at follow-up visits only					
**Treatment-related SAE**	-	-	0.88 (0.69, 1.17)	0.95 (0.72,1.32)	0.96 (0.73,1.32)
**mm Hg SBP above goal, per 5 mm Hg**	-	-	-	0.72 (0.70,0.74)	0.94 (0.93,0.94)
**Number of prior visits with therapeutic inertia**	-	-	-	1.03 (1.01,1.06)	1.03 (1.01,1.06)
**Interaction between race/ethnicity and time**					
Non-Hispanic Black×year	-	-	-	-	0.98 (0.92,1.03)
Hispanic×year	-	-	-	-	1.18 (1.06,1.30)

Model 1 included race/ethnicity and time as the only fixed effects.
Model 2 was adjusted for race and time, in addition to age, sex, education, employment, living with others, insurance status, source of care, smoking status, BMI, depression, statin use, aspirin use, as well as baseline SBP, eGFR, serum potassium, serum sodium, number of antihypertensive medications, prior mTIS, ACEI/ARB, CCB, thiazide diuretic, loop diuretic, beta-blocker, alpha-blocker, and number of non-antihypertensive medications.

Model 3 included variables in Model 2 plus adjustment for follow-up clinical measurements and serious adverse events reported within one month prior of the study visit.
Model 4 included variables in Model 3 plus adjustment for the mm Hg the systolic blood pressure is above the treatment goal and the number of prior study visits with therapeutic inertia.
Model 5 included the same predictors as Model 4 except for the addition of an interaction between race/ethnicity and time.
ACEI: angiotensin converting enzyme inhibitor; ARB: angiotensin II receptor blocker; BMI: body mass index; CCB: calcium channel blocker; CI: confidence interval; eGFR: estimated glomerular filtration rate; mTIS: modified therapeutic intensity score; SAE: serious adverse event; SBP: systolic blood pressure; SPRINT: Systolic Blood Pressure Intervention Trial; VA: Veterans Affairs

© 2021 Zheutlin AR et al. *JAMA Network Open.*

eTable 6. Factors associated with therapeutic inertia among SPRINT participants in the intensive treatment arm

	Odds Ratios (95% CI)				
**Variable**	**Model 1**	**Model 2**	**Model 3**	**Model 4**	**Model 5**
**Non-Hispanic White**	**1 (Reference)**	**1 (Reference)**	**1 (Reference)**	**1 (Reference)**	**1 (Reference)**
**Non-Hispanic Black**	0.94 (0.90, 1.00)	0.96 (0.90, 1.02)	0.94 (0.88, 1.01)	0.99 (0.92,1.05)	0.99 (0.92,1.05)
**Hispanic**	0.86 (0.76, 0.95)	0.87 (0.78, 0.97)	0.89 (0.79, 1.00)	0.99 (0.87,1.10)	0.95 (0.84,1.06)
**Follow-up time** – per one year increase	1.44 (1.41, 1.47)	1.44 (1.41, 1.48)	1.41 (1.37, 1.44)	1.21 (1.17,1.25)	1.20 (1.15,1.25)
**Age –** per 10 year increase	-	1.07 (1.02, 1.11)	1.09 (1.04, 1.14)	1.10 (1.06,1.15)	1.10 (1.06,1.15)
**Female sex**	-	1.01 (0.95, 1.07)	1.02 (0.96, 1.09)	1.04 (0.98,1.11)	1.04 (0.98,1.11)
**Education**					
High school graduate or lower	**1 (Reference)**	**1 (Reference)**	**1 (Reference)**	**1 (Reference)**	**1 (Reference)**
Post High school training/education	-	1.05 (0.98, 1.11)	1.05 (0.98, 1.12)	1.03 (0.97,1.10)	1.03 (0.97,1.10)
**Full-time employment**	-	1.01 (0.92, 1.10)	1.00 (0.91, 1.11)	0.97 (0.89,1.08)	0.97 (0.89,1.08)
**Retired**	-	0.96 (0.88, 1.03)	0.95 (0.88, 1.03)	0.94 (0.88,1.03)	0.94 (0.88,1.02)
**Lives with others**	-	1.00 (0.95, 1.06)	1.00 (0.94, 1.07)	1.01 (0.95,1.07)	1.01 (0.95,1.07)
**Insurance status**					
Uninsured	**1 (Reference)**	**1 (Reference)**	**1 (Reference)**		
Medicare/Medicaid	-	1.03 (0.96, 1.11)	1.04 (0.96, 1.12)	1.04 (0.96,1.11)	1.04 (0.96,1.11)
Private	-	1.02 (0.96, 1.08)	1.02 (0.96, 1.08)	1.01 (0.95,1.07)	1.01 (0.95,1.07)
VA	-	0.94 (0.87, 1.01)	0.94 (0.87, 1.02)	0.95 (0.88,1.02)	0.95 (0.88,1.02)
**Source of care**					
No usual source	**1 (Reference)**	**1 (Reference)**	**1 (Reference)**	**1 (Reference)**	**1 (Reference)**
Hospital/clinic	-	1.18 (1.02, 1.37)	1.16 (1.00, 1.35)	1.14 (1.00,1.33)	1.14 (0.99,1.33)
Community health center	-	1.17 (0.99, 1.35)	1.13 (0.97, 1.33)	1.11 (0.95,1.30)	1.11 (0.96,1.29)
**Smoking status**					
Never	**1 (Reference)**	**1 (Reference)**	**1 (Reference)**	**1 (Reference)**	**1 (Reference)**
Current	-	0.93 (0.84, 1.01)	0.92 (0.83, 1.00)	0.95 (0.87,1.04)	0.95 (0.86,1.03)
Former	-	1.02 (0.97, 1.08)	1.01 (0.96, 1.08)	1.01 (0.95,1.07)	1.01 (0.95,1.07)
**BMI, kg/m²** – per 5 kg/m² increase	-	0.98 (0.95, 1.00)	0.96 (0.94, 0.99)	0.96 (0.94,0.99)	0.96 (0.94,0.99)
**SBP –** per 10 mm Hg increase	-	1.00 (1.00,1.00)	0.99 (0.99,0.99)	0.95 (0.87,1.04)	0.95 (0.86,1.03)
**eGFR, mL/min/1.73m²** – per 10 mL/min/1.73m2 increase	-	1.01 (0.99, 1.03)	0.91 (0.90, 0.93)	1.01 (0.95,1.07)	1.01 (0.95,1.07)
**Serum potassium, mmol/L –** per 0.5 mmol/L increase	-	0.98 (0.92, 1.05)	1.06 (1.00, 1.12)	1.05 (1.00,1.11)	1.05 (1.00,1.11)
**Serum sodium, mEq/L –** per 5 mEq/L increase	-	0.98 (0.89, 1.09)	0.99 (0.90, 1.07)	0.99 (0.90,1.07)	0.99 (0.90,1.07)
**Depression**	-	0.97 (0.90, 1.03)	0.97 (0.90, 1.04)	0.99 (0.93,1.06)	0.99 (0.93,1.06)
**Number of antihypertensive medications**	-	1.02 (0.95, 1.09)	1.07 (1.00, 1.14)	1.04 (0.98,1.11)	1.04 (0.98,1.11)
**Prior mTIS**	-	1.04 (1.01, 1.08)	1.13 (1.08, 1.17)	1.14 (1.09,1.18)	1.14 (1.09,1.18)
**ACEI or ARB**	-	0.99 (0.91, 1.07)	0.90 (0.83, 0.97)	0.94 (0.87,1.02)	0.94 (0.87,1.02)
**CCB**	-	1.03 (0.94, 1.11)	0.94 (0.87, 1.02)	0.91 (0.85,0.99)	0.91 (0.85,0.99)
**Thiazide diuretic**	-	1.02 (0.93, 1.13)	1.16 (1.08, 1.27)	1.17 (1.09,1.27)	1.16 (1.09,1.26)
**Loop diuretic**	-	1.02 (0.89, 1.16)	1.04 (0.92, 1.17)	1.05 (0.95,1.18)	1.05 (0.94,1.18)
**Beta-blocker**	-	0.99 (0.91, 1.07)	0.92 (0.86, 1.00)	0.95 (0.89,1.03)	0.95 (0.88,1.03)
**Alpha-blocker**	-	1.01 (0.93, 1.10)	0.85 (0.76, 0.94)	0.86 (0.78,0.94)	0.86 (0.78,0.94)
**Number of non-antihypertensive medications**	-	0.99 (0.98, 1.00)	1.00 (0.99, 1.00)	1.00 (0.99,1.00)	1.00 (0.99,1.00)
**Current medication**					
Statin	-	1.00 (0.95, 1.05)	0.98 (0.93, 1.05)	0.99 (0.94,1.04)	0.99 (0.94,1.04)
Aspirin	-	0.98 (0.92, 1.03)	0.97 (0.92, 1.03)	0.96 (0.91,1.01)	0.96 (0.91,1.01)
Collected at follow-up visits only					
**Treatment-related SAE**	-	-	0.99 (0.83, 1.19)	0.99 (0.83,1.17)	0.99 (0.83,1.17)
**mm Hg SBP above goal, per 5 mm Hg**	-	-	-	0.89 (0.88,0.90)	0.98 (0.97,0.98)
**Number of prior visits with therapeutic inertia**				1.09 (1.07,1.10)	1.09 (1.08,1.11)
**Interaction between race/ethnicity and time**					
Non-Hispanic Black×year	-	-	-	-	1.00 (0.95,1.05)
Hispanic×year	-	-	-	-	1.13 (1.02,1.23)

Model 1 included race/ethnicity and time as the only fixed effects.
Model 2 was adjusted for race and time, in addition to age, sex, education, employment, living with others, insurance status, source of care, smoking status, BMI, depression, statin use, aspirin use, as well as baseline SBP, eGFR, serum potassium, serum sodium, number of antihypertensive medications, prior mTIS, ACEI/ARB, CCB, thiazide diuretic, loop diuretic, beta-blocker, alpha-blocker, and number of non-antihypertensive medications.

© 2021 Zheutlin AR et al. *JAMA Network Open.*

Model 3 included variables in Model 2 plus adjustment for follow-up clinical measurements and serious adverse events reported within one month prior of the study visit.<br>Model 4 included variables in Model 3 plus adjustment for the mm Hg the systolic blood pressure is above the treatment goal and the number of prior study visits with therapeutic inertia.<br>Model 5 included the same predictors as Model 4 except for the addition of an interaction between race/ethnicity and time.<br>ACEI: angiotensin converting enzyme inhibitor; ARB: angiotensin II receptor blocker; BMI: body mass index; CCB: calcium channel blocker; CI: confidence interval; eGFR: estimated glomerular filtration rate; mTIS: modified therapeutic intensity score; SAE: serious adverse event; SBP: systolic blood pressure; SPRINT: Systolic Blood Pressure Intervention Trial; VA: Veterans Affairs

© 2021 Zheutlin AR et al. *JAMA Network Open.*

eFigure 1. Boxplots and histograms of mm Hg over BP goal stratified by study arm and race/ethnicity at 12 months.

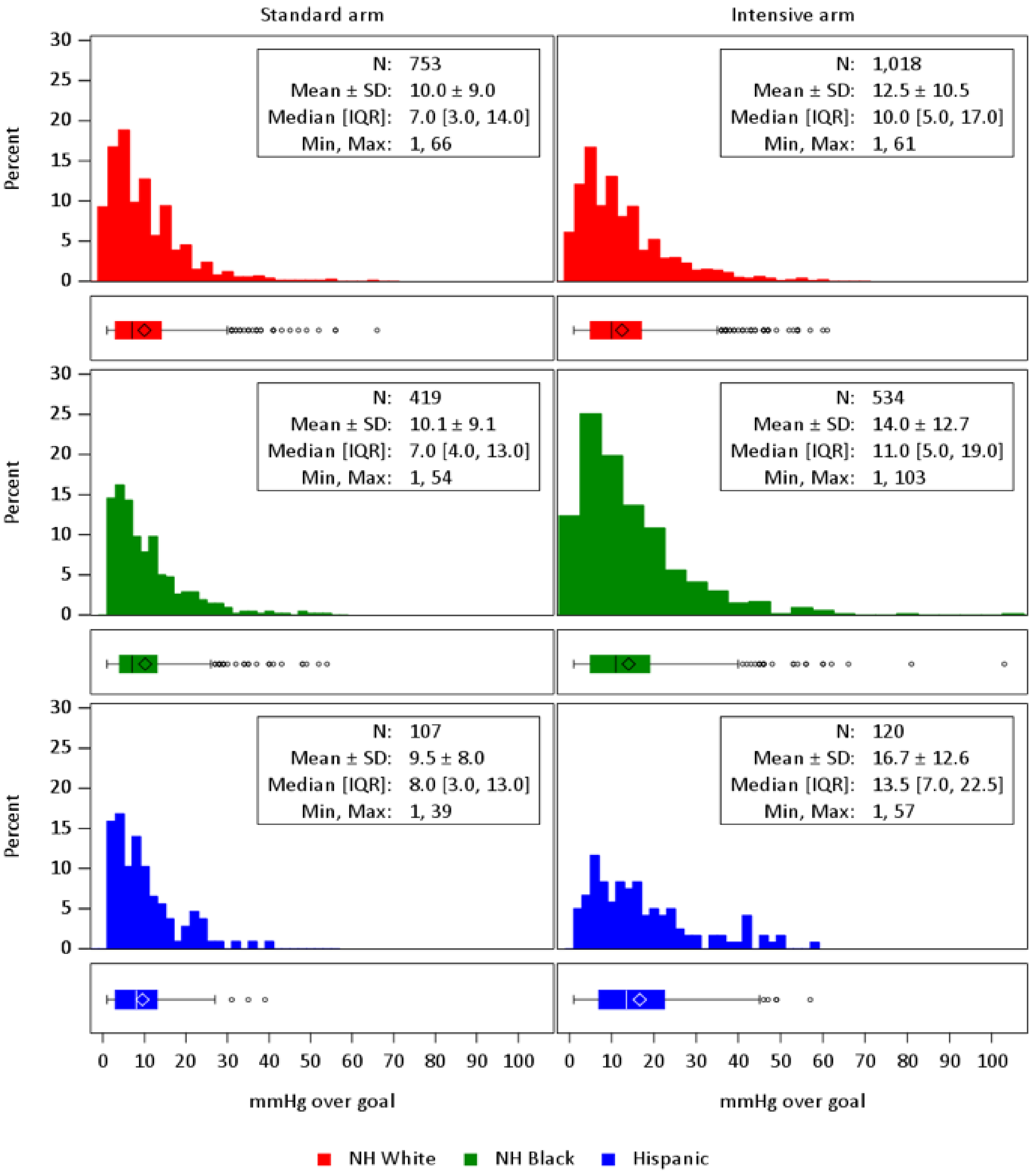

NH= Non-Hispanic

© 2021 Zheutlin AR et al. *JAMA Network Open.*

eFigure 2. Boxplots and histograms of mm Hg over BP goal stratified by study arm and race/ethnicity at 24 months.

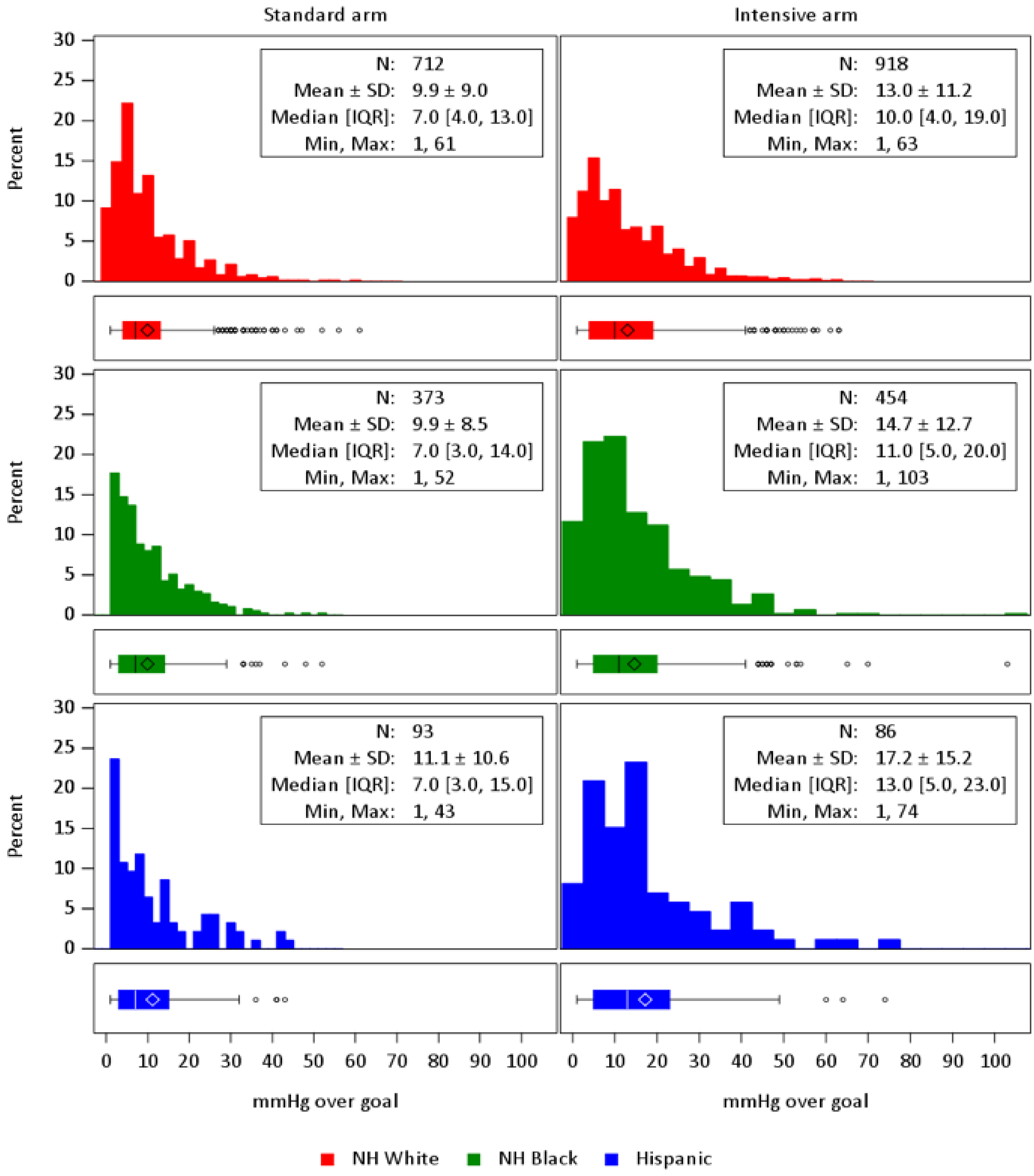

NH= Non-Hispanic

© 2021 Zheutlin AR et al. *JAMA Network Open.*

eFigure 3. Boxplots and histograms of mm Hg over BP goal stratified by study arm and race/ethnicity at 36 months.

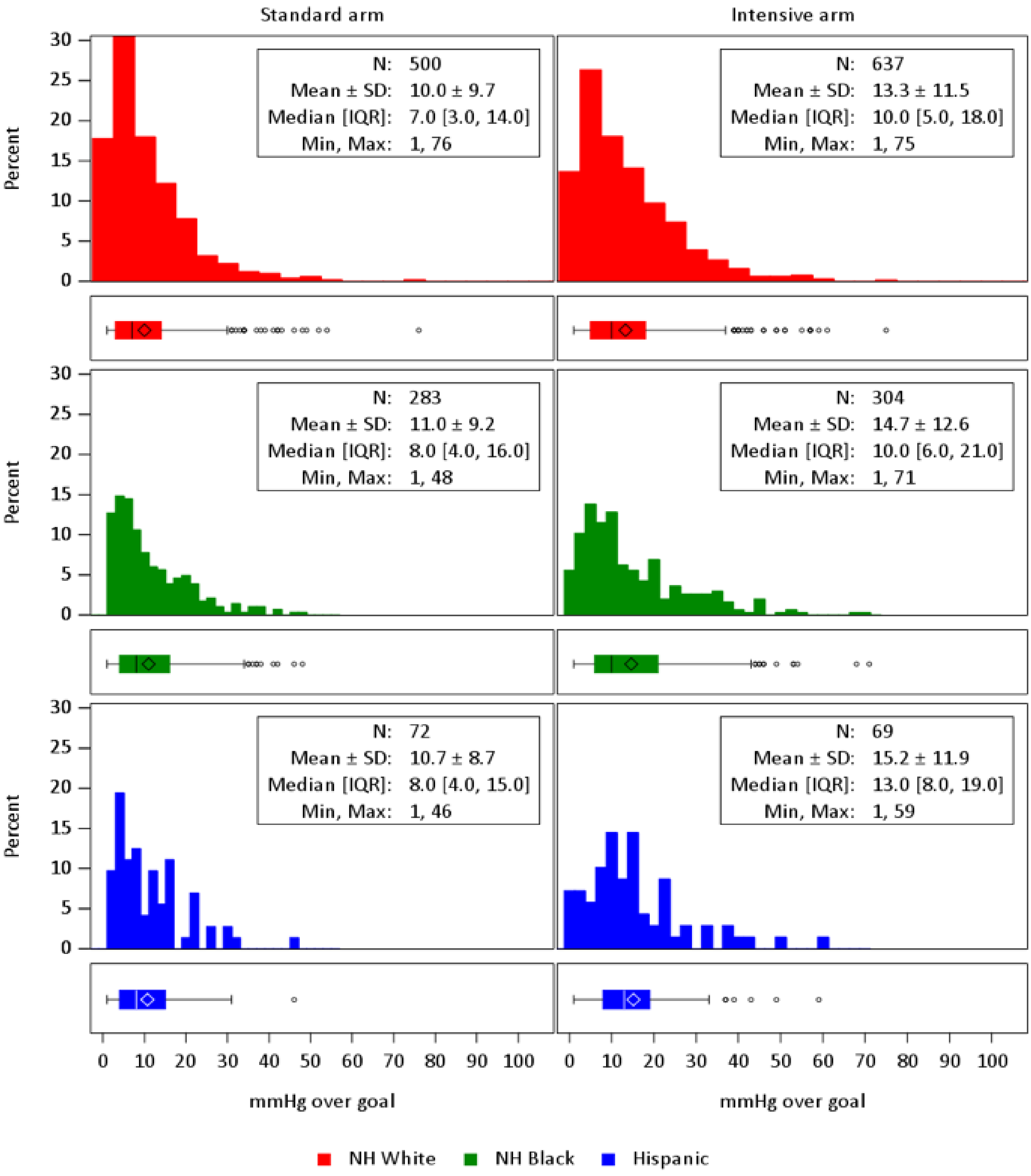

NH= Non-Hispanic

© 2021 Zheutlin AR et al. *JAMA Network Open.*

eFigure 4. Boxplots and histogramsof mm Hg over BP goal stratified by study arm and race at 48 months.

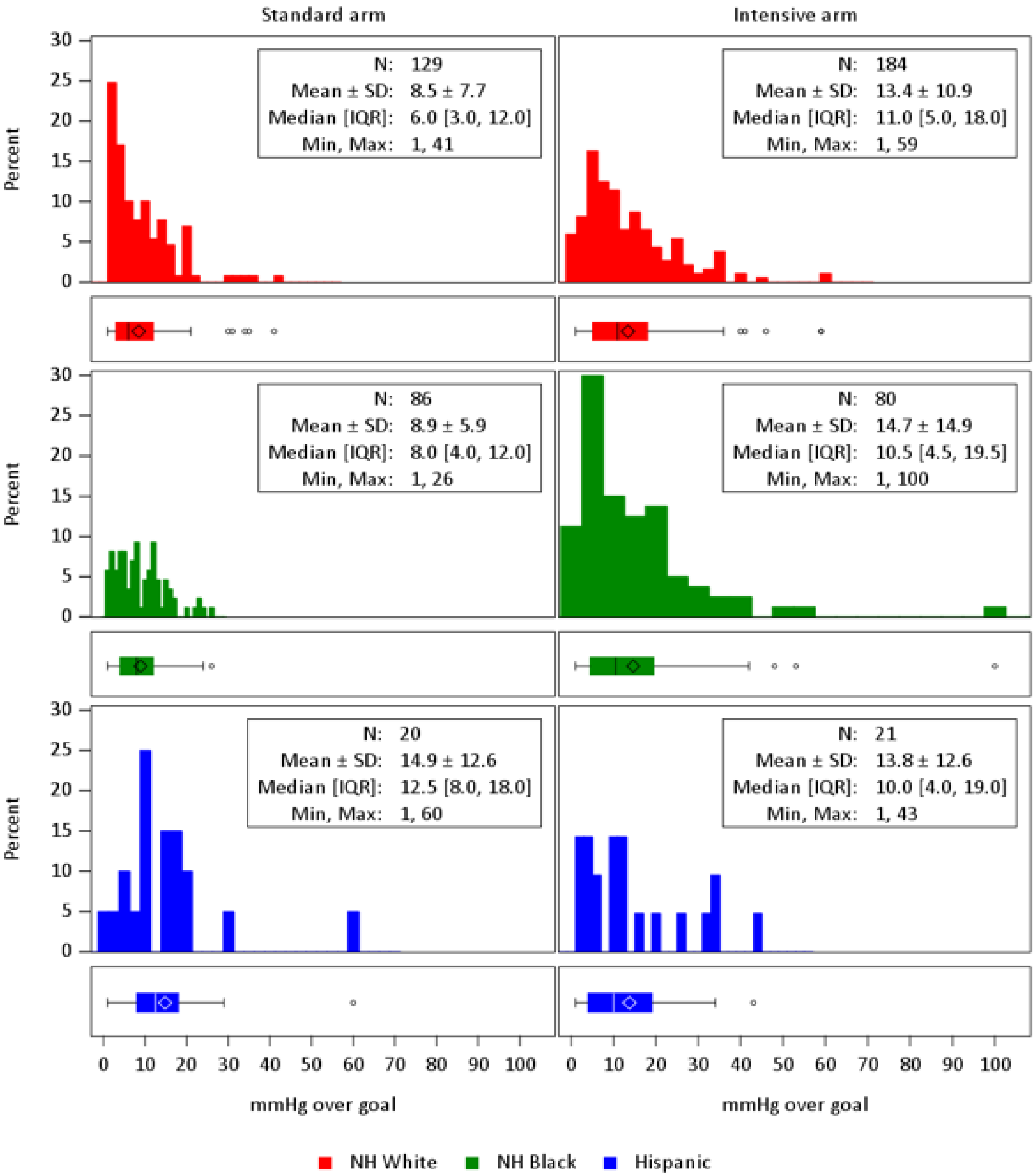

NH= Non-Hispanic

© 2022 Zheutlin AR et al. *JAMA Network Open.*

eFigure 5. Factors associated with therapeutic inertia among SPRINT participants by race/ethnicity in the standard treatment arm

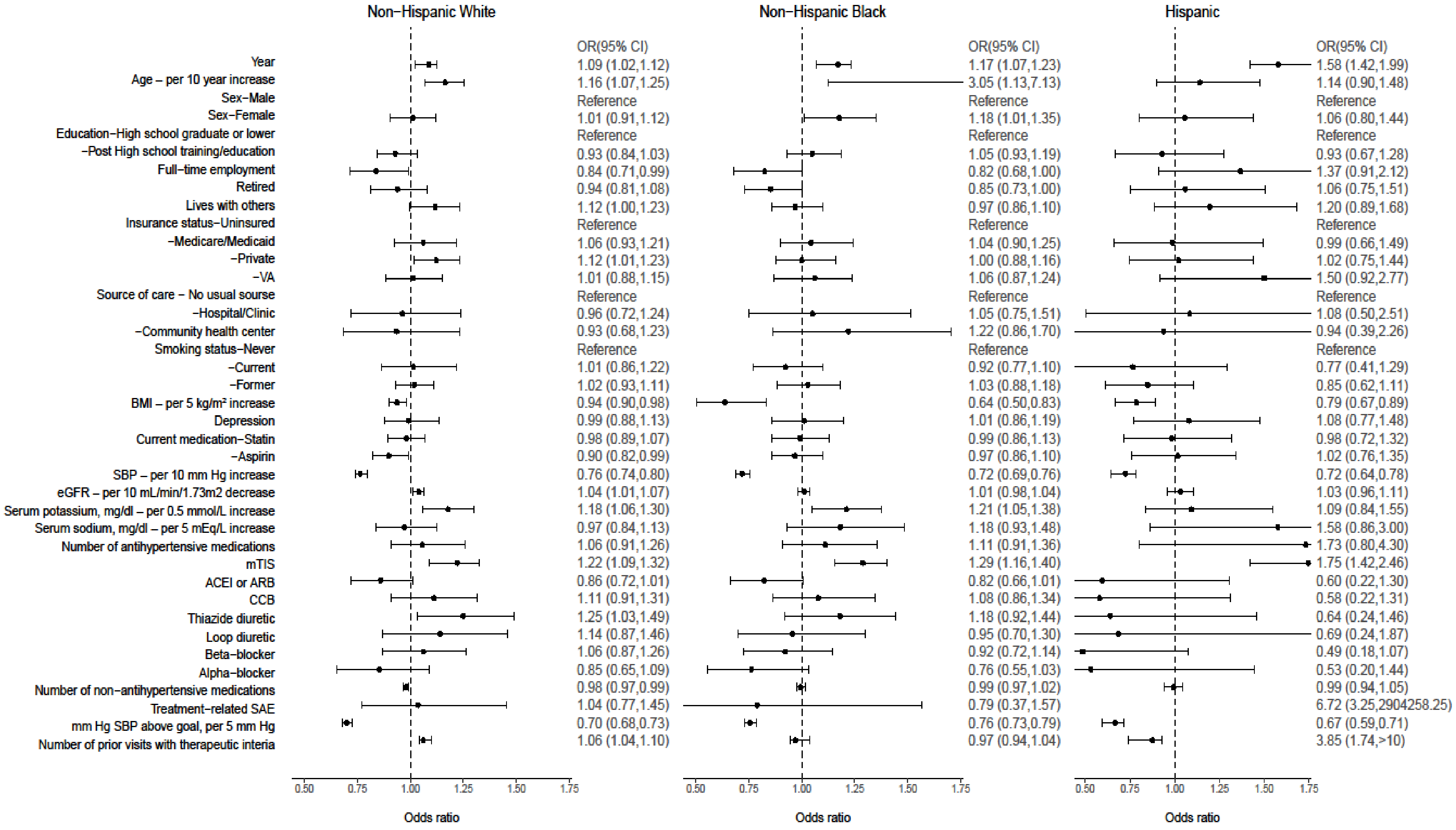

Odds ratios for therapeutic inertia among SPRINT participants in the standard arm, stratified by self-reported race/ethnicity. Calculations are based off of Model 4, which includes adjustment for time, in addition to age, sex, education, employment, living with others, insurance status, source of care, smoking status, BMI, depression, statin use, aspirin use, as well as most recent visit SBP, eGFR, serum potassium, serum sodium, number of antihypertensive medications, prior mTIS, ACEI/ARB, CCB, thiazide diuretic, loop diuretic, beta-blocker, alpha-blocker, number of non-antihypertensive medications, serious adverse events reported within one month prior of the study visit, the mm Hg the systolic blood pressure is above the treatment goal, and the number of prior study visits with therapeutic inertia. *Abbreviations:* ACEI: angiotensin converting enzyme inhibitor; ARB: angiotensin II receptor blocker; BMI: body mass index; CCB: calcium channel blocker; CI: confidence interval; eGFR: estimated glomerular filtration rate; mTIS: modified therapeutic intensity score; OR: odds ratio; SAE: serious adverse event; SBP: systolic blood pressure; SPRINT: Systolic Blood Pressure Intervention Trial; VA: Veterans Affairs.

© 2022 Zheutlin AR et al. *JAMA Network Open.*

eFigure 6. Factors associated with therapeutic inertia among SPRINT participants by race/ethnicity in the intensive treatment arm

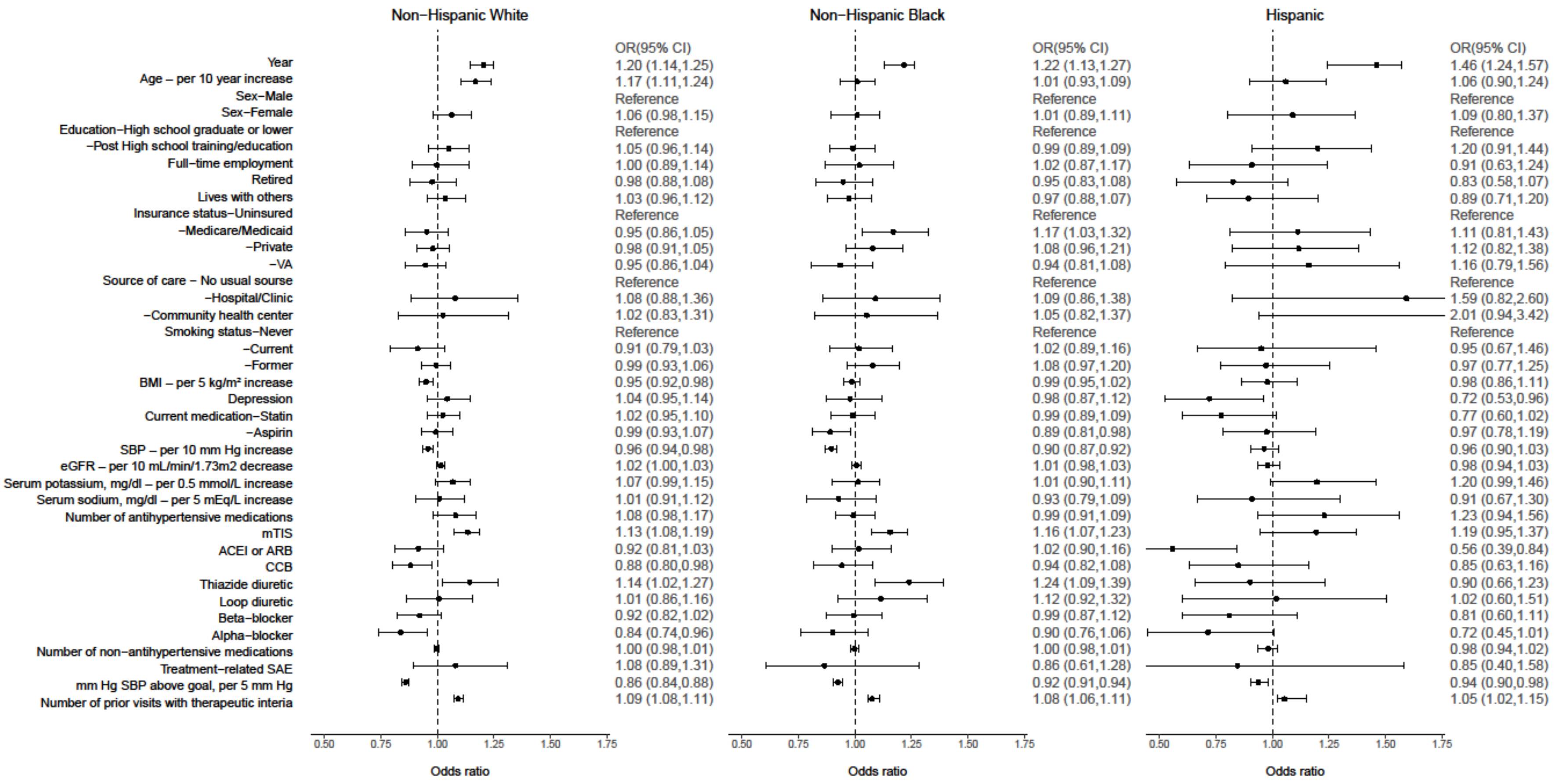

Odds ratio for therapeutic inertia among SPRINT participants in the intensive arm, stratified by self-reported race/ethnicity. Calculations are based off of Model 4, which includes adjustment for time, in addition to age, sex, education, employment, living with others, insurance status, source of care, smoking status, BMI, depression, statin use, aspirin use, as well as most recent visit SBP, eGFR, serum potassium, serum sodium, number of antihypertensive medications, prior mTIS, ACEI/ARB, CCB, thiazide diuretic, loop diuretic, beta-blocker, alpha-blocker, number of non-antihypertensive medications, serious adverse events reported within one month prior of the study visit, the mm Hg the systolic blood pressure is above the treatment goal, and the number of prior study visits with therapeutic inertia. *Abbreviations:* ACEI: angiotensin converting enzyme inhibitor; ARB: angiotensin II receptor blocker; BMI: body mass index; CCB: calcium channel blocker; CI: confidence interval; eGFR: estimated glomerular filtration rate; mTIS: modified therapeutic intensity score; OR: odds ratio; SAE: serious adverse event; SBP: systolic blood pressure; SPRINT: Systolic Blood Pressure Intervention Trial; VA: Veterans Affairs.

© 2022 Zheutlin AR et al. *JAMA Network Open.*

eReferences

1. Berlowitz DR. Clinical inertia and the 2017 ACA/AHA guideline. *J Clin Hypertens*. 2018;20(10):1392-1394. doi:10.1111/jch.13373
2. Rose AJ, Berlowitz DR, Manze M, Orner MB, Kressin NR. Comparing methods of measuring treatment intensification in hypertension care. *Circ Cardiovasc Qual Outcomes*. 2009;2(4):385-391. doi:10.1161/CIRCOUTCOMES.108.838649
3. Whelton PK, Carey RM, Aronow WS, et al. 2017 ACC/AHA/AAPA/ABC/ACPM/ AGS/APhA/ASH/ASPC/NMA/PCNA Guideline for the Prevention, Detection, Evaluation, and Management of High Blood Pressure in Adults: A Report of the American College of Cardiology/American Heart Association Task Force on Clinical P. *Circulation*. 2018;71(17):e127-e248. doi:10.1016/j.jacc.2017.11.006
4. von Elm E, Altman DG, Egger M, et al. Strengthening the Reporting of Observational Studies in Epidemiology (STROBE) in the International Journal of Medical Students. *BMJ*. 2007;335(7624):806-808. doi:10.1136/bmj.39335.541782.AD

© 2022 Zheutlin AR et al. *JAMA Network Open.*
